# Remote Actuation Systems for Fully Wearable Assistive Devices: Requirements, Selection, and Optimization for Out-of-the-Lab Application of a Hand Exoskeleton

**DOI:** 10.3389/frobt.2020.596185

**Published:** 2021-01-28

**Authors:** Jan Dittli, Urs A. T. Hofmann, Tobias Bützer, Gerwin Smit, Olivier Lambercy, Roger Gassert

**Affiliations:** ^1^Rehabilitation Engineering Laboratory, Department of Health Sciences and Technology, ETH Zurich, Zurich, Switzerland; ^2^Department of BioMechanical Engineering, Delft University of Technology, Delft, Netherlands

**Keywords:** soft robotics, hand exoskeleton, remote actuation, cable-driven, Bowden cable, wearable robot, assistive device, out-of-the-lab

## Abstract

Wearable robots assist individuals with sensorimotor impairment in daily life, or support industrial workers in physically demanding tasks. In such scenarios, low mass and compact design are crucial factors for device acceptance. Remote actuation systems (RAS) have emerged as a popular approach in wearable robots to reduce perceived weight and increase usability. Different RAS have been presented in the literature to accommodate for a wide range of applications and related design requirements. The push toward use of wearable robotics in out-of-the-lab applications in clinics, home environments, or industry created a shift in requirements for RAS. In this context, high durability, ergonomics, and simple maintenance gain in importance. However, these are only rarely considered and evaluated in research publications, despite being drivers for device abandonment by end-users. In this paper, we summarize existing approaches of RAS for wearable assistive technology in a literature review and compare advantages and disadvantages, focusing on specific evaluation criteria for out-of-the-lab applications to provide guidelines for the selection of RAS. Based on the gained insights, we present the development, optimization, and evaluation of a cable-based RAS for out-of-the-lab applications in a wearable assistive soft hand exoskeleton. The presented RAS features full wearability, high durability, high efficiency, and appealing design while fulfilling ergonomic criteria such as low mass and high wearing comfort. This work aims to support the transfer of RAS for wearable robotics from controlled lab environments to out-of-the-lab applications.

## 1. Introduction

The field of wearable robotics has received increasing interest over the past years, in particular with the introduction of soft materials and technologies (Chu and Patterson, 2018; Walsh, 2018). Thanks to their inherent compliance allowing to closely mimic, follow, or support a user's motion, soft wearable robots may offer unique beneficial properties in terms of comfort, safety, and efficiency compared to their rigid counterparts (Chiaradia et al., 2019; Sanchez-Villamanan et al., 2019). As such, they are promising solutions for applications as assistive devices supporting individuals with impairment in therapy or activities of daily living (ADLs) or to facilitate physically demanding tasks in industrial work. Most application scenarios targeting daily use of such wearable robots require full wearability besides inherent softness (Chu and Patterson, 2018). In the design of fully wearable (i.e., untethered) devices, small size and especially low mass and inertia while providing high output forces or torques are crucial factors (Sarac et al., 2019). Perceived carried weight on the extremities was identified as the most critical design property for users to accept or abandon their devices in a study with 242 prosthesis users (Biddiss et al., 2007). When mass and volume constraints cannot be met, remote actuation systems (RAS) are an excellent alternative to minimize carried weight on extremities (Veale and Xie, 2016). Typically, a RAS consists of an actuation unit, a transmission system, and an output. The actuation unit provides mechanical power, which is transmitted by the transmission system. The output transforms the power into the motion and force required by the wearable assistive device. While mass and volume of the overall system increase due to the additional components required for power transmission, remote actuation reallocates the actuator mass away from the extremities to more proximal body parts, e.g., the trunk, and consequently reduces perceived load and inertial effects.

Many different remote actuation principles have been described in the literature for wearable devices (Bos et al., 2016; Veale and Xie, 2016; Manna and Dubey, 2018), all featuring different advantages and drawbacks for wearable applications. In this large design space, selecting and optimizing appropriate RAS can be challenging due to the wide range of application scenarios for wearable robots. The requirements vary depending on the wearable device to actuate, the body functions to be supported, and the constraints linked to the intended use case. Wearable assistive devices that are expected to improve the quality of life of people with sensorimotor impairment by functionally supporting wearers in ADLs (e.g., Yap et al., 2016; Haufe et al., 2019) represent one of the most common application scenarios for RAS (Bos et al., 2016). Low mass and volume on the supported extremities are especially relevant for applications of wearable assistive devices since the extremities of users with sensorimotor impairment are commonly weak (Chae et al., 2002; Ada et al., 2006). Although a large number of remote actuation system (RAS) for wearable assistive devices have been presented, many of these RAS still struggle with full wearability due to often bulky designs. Besides, as many research projects strive for the transfer of wearable assistive robots out of the controlled lab environments toward longitudinal testing in home environments or clinics (Reinkensmeyer, 2019), additional key requirements for RAS emerge that are often not fulfilled by current wearable assistive devices, resulting in low device adoption by end-users (Chu and Patterson, 2018). In particular, a major challenge in the transfer of wearable assistive technologies to such out-of-the-lab applications is durability. This aspect is rarely evaluated and reported in research publications, although highly significant for longitudinal applications and testing (Jeong et al., 2020). Furthermore, properties influencing wearability, e.g., overall mass and volume, and device ergonomics (e.g., wearing comfort, and heat and noise emission) become even more important. By considering these specific requirements throughout the entire design process of a RAS, starting from the selection of the working principle and its mechanical components up to the performance evaluation, we expect to move wearable robots closer to out-of-the-lab applications.

The objective of this paper is to provide an overview of RAS for wearable assistive devices and discuss existing solutions in view of out-of-the-lab applications. In particular, design requirements such as durability, efficiency, ergonomics, and maintenance are considered to identify the most suitable RAS to actuate a fully wearable assistive robot. Based on the gained insights, we revisit and optimize a previously presented RAS (Hofmann et al., 2018) for long-term, independent out-of-the-lab use with a fully wearable hand exoskeleton for assistance during ADLs in people with hand neuromotor impairment (Bützer et al., 2020), representing a typical design case of a wearable assistive device requiring an efficient, lightweight, and soft RAS. Finally, we present the performance evaluation of the developed RAS with regard to durability and ergonomics.

## 2. Materials and Methods

### 2.1. Literature Review

A review of RAS for wearable assistive devices presented in the literature was conducted to identify the most common working principles. The search was performed online on scholar.google.com with the keywords “(torque transmission or force transmission or remote actuation or remotely actuated or remote actuator) and (portable or wearable) and (device or robot or system)” in May 2020. Wearable devices presented in those papers were screened for their application scenario and actuation principle. Publications making use of direct drive systems or aiming at intended applications other than fully wearable assistive robotics were excluded. For the qualitative analysis, the RAS were divided into the three main subunits (actuation unit, transmission system, and output). Additionally, the RAS were categorized by the type of transmission principle (pneumatic, hydraulic, and cable-based). Emerging actuator technologies such as heat-driven shape memory alloys (SMA), electroactive polymers (EAP), or piezoelectric motors were not analyzed in this review due to their currently still low technology readiness and major limitations in terms of wearability, output power, bandwidth, need for dedicated electronics, and applicability in out-of-the-lab use (Veale and Xie, 2016; Manna and Dubey, 2018; Zhu et al., 2020). Furthermore, hybrid approaches combining pneumatic and cable-based transmission systems were excluded (Jiang et al., 2018; Stilli et al., 2018; Gerez et al., 2020). While uniting transmission-specific advantages, the increased complexity and need for multiple actuation systems limit the application in fully wearable robotics. In total, 81 manuscripts describing RAS were included in the qualitative review.

The underlying working principles of pneumatic, hydraulic, and cable-based RAS and their technical implementation are summarized and discussed in the following. Afterward, we examine the applicability, advantages, and disadvantages to rate the RAS principles with respect to the specific context of out-of-the-lab applications of wearable assistive devices.

#### 2.1.1. Pneumatic Transmissions

Pneumatics is one of the most commonly used RAS principles for wearable assistive devices, mainly for upper-limb applications (He et al., 2005; Sasaki et al., 2005; Takahashi et al., 2005; Takahashi et al., 2020; Costa and Caldwell, 2006; Gordon et al., 2006; Balasubramanian et al., 2008; Beyl et al., 2008; Jia-Fan et al., 2008; Ino et al., 2009; Vanderhoff and Kim, 2009; Xing et al., 2009; Hurst and Aw, 2011; Bae and Moon, 2012; Heo et al., 2013; Huang and Chen, 2013; Tjahyono et al., 2013; Noda et al., 2014; Park et al., 2014a,b; Patar et al., 2014; Yap et al., 2015; Zhao et al., 2015; Haghshenas-Jaryani et al., 2016; Chen et al., 2017; Sun et al., 2017; Yun Y. et al., 2017; Zhou et al., 2019; Ge et al., 2020; Hong et al., 2020). In the actuation unit, pneumatic energy is stored in form of highly pressurized air or generated by pumps powered by batteries. Usually, valves and regulators control airflow and pressure through pneumatic hoses (tubes) made from polyurethane or polyethylene (PE). At the output, pneumatic actuators transform the power provided as pressurized air into the mechanical power required by the wearable assistive device, i.e., force or torque, and linear or rotary motion.

Small and lightweight pneumatic hoses can transmit high mechanical power by pressurized air (typical pressure range from 0.6 to 1 MPa). The compliance of air is advantageous for safe interaction between human and robot. At low gas speeds and flow rates, the pressure drops along pneumatic hoses are low and not influenced by changing bending angles of the transmission system. In return, the compliance limits the achievable force/position bandwidth. Since air can be released into the surrounding, there is no need for return or collection systems. However, potential energy is released together with the pressurized air after performing work, negatively impacting the efficiency of the RAS. Long transmission lines increase the dead volume and therefore reduce efficiency. At high pressures, hoses can burst, potentially causing severe injuries.

Most frequently, conventional pressure generators or external compressed air supplies (e.g., stationary air supply systems as found in many laboratories) have been used as power sources at the input (He et al., 2005; Sasaki et al., 2005; Takahashi et al., 2005; Gordon et al., 2006; Beyl et al., 2008; Heo et al., 2013; Huang and Chen, 2013; Park et al., 2014b; Patar et al., 2014; Zhao et al., 2015; Haghshenas-Jaryani et al., 2016; Chen et al., 2017; Sun et al., 2017; Zhou et al., 2019; Hong et al., 2020). Alternatively, compressors based on metal hydrides (MH) have been presented (Ino et al., 2009; Vanderhoff and Kim, 2009). MHs are capable of storing a large amount of hydrogen, which is released from or bound to the metal in a reversible chemical reaction as soon as the MH is heated or cooled. Other RAS either used miniature pneumatic pumps (Lucas et al., 2004; Yun Y. et al., 2017; Ge et al., 2020) or tanks storing pressurized air or liquid gas (Noda et al., 2014; Park et al., 2014b; Luo et al., 2015) to provide pneumatic pressure.

At the output, pneumatic artificial muscles (PAM) are one possible solution offering very high power at low mass. PAM are often configured in pairs (agonist and antagonist) (Gordon et al., 2006; Beyl et al., 2008; Jia-Fan et al., 2008; Noda et al., 2014) or combined with springs for bidirectional force control (He et al., 2005; Heo et al., 2013), since an individual PAM can only provide unidirectional forces by contracting upon inflation, similar to human muscles. In contrast to PAM, pneumatic bellows extend upon activation (Ino et al., 2009; Yun Y. et al., 2017; Zhou et al., 2019). Recently, soft pneumatic actuators (SPA) made from silicone-like materials and designed to follow complex motion patterns received increasing interest (Yap et al., 2015; Zhao et al., 2015; Haghshenas-Jaryani et al., 2016; Agarwal et al., 2017; Sun et al., 2017; Hong et al., 2020). SPA with variable stiffness can accurately reproduce motion patterns without rigid mechanical support structures like joints or links (Yap et al., 2015; Zhao et al., 2015; Haghshenas-Jaryani et al., 2016; Agarwal et al., 2017; Sun et al., 2017). Double- and single-acting pneumatic cylinders are a further common type of output (Lucas et al., 2004; Takahashi et al., 2005; Bae and Moon, 2012; Huang and Chen, 2013; Patar et al., 2014). Cylinders can exert high bidirectional linear forces but have a limited stroke length and suffer from relatively high static friction resulting from integrated sealings.

#### 2.1.2. Hydraulic Transmissions

Hydraulic power transmissions have been used frequently in RAS (Takemura et al., 2005; Kargov et al., 2008; Pylatiuk et al., 2009; Kaminaga et al., 2010; Ohnishi et al., 2013; Bechet and Ohnishi, 2014; Lee, 2014; Smit et al., 2014b; Polygerinos et al., 2015; Ouyang et al., 2016; Bos et al., 2020; Chen et al., 2020). In contrast to pneumatic RAS, which are usually limited to pressure control, pressure and flow can be generated and controlled at the input of hydraulic RAS. Hydraulic hoses filled with an incompressible fluid connect the actuation unit to the output and transmit the generated power. At the output, hydraulic actuators transduce the hydraulic power into the required mechanical power. Hydraulic RAS feature high controllability and bandwidth. The transmission system is often stiffer compared to other RAS principles, primarily if operated at high pressures (typical pressure range from 1 to 10 MPa). Furthermore, return systems are required for the fluid.

Since liquids are nearly incompressible, they cannot be pre-pressurized to store energy in a tank. Therefore, hydraulic pressure and flow have to be generated in the actuation unit. Most hydraulic systems use hydraulic pumps in combination with valves to control the flow through the hoses (Kargov et al., 2008; Pylatiuk et al., 2009; Kaminaga et al., 2010; Lee, 2014; Polygerinos et al., 2015; Ouyang et al., 2016). Hydraulic cylinders are a different common approach to generate pressure and flow. Linear or rotary electromagnetic motors (Ohnishi et al., 2013; Bechet and Ohnishi, 2014; Chen et al., 2020) or body power (Smit et al., 2014b; Bos et al., 2020) have been used to drive the piston of conventional hydraulic cylinders.

The most common types of hydraulic outputs are single- and double-acting cylinders combined with hydraulic cylinders driven by electric motors (Ohnishi et al., 2013; Bechet and Ohnishi, 2014; Bos et al., 2020) or pumps (Lee, 2014; Ouyang et al., 2016) in the actuation unit. Bellow actuators (Kargov et al., 2008; Pylatiuk et al., 2009), implementations of hydraulic artificial muscles (HAM) (Takemura et al., 2005), and soft hydraulic actuators (SHA) (Polygerinos et al., 2015; Chen et al., 2020) have been integrated into hydraulic-based RAS. The working principles of the different outputs cylinders, bellows, HAM, and SHA are similar to their equivalent pneumatic version.

#### 2.1.3. Cable-Based Transmissions

In cable-based RAS, a rope or a cable mechanically connects the actuation unit to the output. Most of the reviewed RAS use Bowden cables consisting of a cable guided in a sheath (Letier et al., 2006; Veneman et al., 2007; Kong et al., 2009; Wang et al., 2009; Vitiello et al., 2013; Agarwal et al., 2015; Asbeck et al., 2015; Bartenbach et al., 2015; Cempini et al., 2015; In et al., 2015; Nycz et al., 2015, 2016; Cappello et al., 2016; Choi et al., 2016; Choi et al., 2019; Dinh et al., 2016; Kalantari and Ghaffari, 2016; Norman et al., 2016; Xiloyannis et al., 2016, 2019; Blumenschein et al., 2017; Jeong and Cho, 2017; Popov et al., 2017; Yun S.-S. et al., 2017; Randazzo et al., 2018; Yi et al., 2018; Burns et al., 2019; Dwivedi et al., 2019; Tran et al., 2020). Electrical motors are most commonly used to actuate the cable transmission. As an output, guide rails or winches are often sufficient, resulting in lightweight and compact designs (Chiri et al., 2009; In et al., 2015; Nycz et al., 2015, 2016; Jeong and Cho, 2017).

Bowden-cable-based RAS can easily provide unidirectional motion to the wearable assistive device by pulling on the transmission cable. To achieve bidirectional transmission, either single rods allowing to transmit pushing forces (push-pull configuration, e.g., Randazzo et al., 2018) or cables configured in pairs (pull–pull configuration, e.g., Hofmann et al., 2018) can be used. Bowden-cable-based transmissions often suffer from backlash and friction losses depending on the bending angle of the transmission system (Agarwal et al., 2015; Nycz et al., 2016; Jeong and Cho, 2020). Consequently, accurate force, torque, or position control have mostly been achieved by placing sensors at the output (Letier et al., 2006; Agarwal et al., 2015; Asbeck et al., 2015). Alternatively, in Hofmann et al. (2018) and Jeong and Cho (2017), bending angle sensors combined with dynamic feed-forward friction compensation were presented, such that no sensors were needed at the output. Often, series elastic elements (SEE) have been used to measure the force transmitted by Bowden cables at the output (Agarwal et al., 2015; Blumenschein et al., 2017; Marconi et al., 2019). SEE have also been used to pretension the wires and make the transmission more compliant (Veneman et al., 2007).

Actuation units of cable-based transmission systems are mostly composed of rotational electromagnetic motors in combination with gears (Veneman et al., 2007; Asbeck et al., 2015; Nycz et al., 2015; Xiloyannis et al., 2016, 2019; Blumenschein et al., 2017; Park et al., 2019; Rose and O'Malley, 2019), pulleys (Vitiello et al., 2013; Choi et al., 2019), lead screws (Cempini et al., 2015), or winches (Letier et al., 2006; Agarwal et al., 2015; In et al., 2015; Cappello et al., 2016; Dinh et al., 2016; Kalantari and Ghaffari, 2016; Xiloyannis et al., 2016; Jeong and Cho, 2017; Thielbar et al., 2017; Tran et al., 2020). Alternatively, linear motors and servo motors, directly attached to the cable, have been used (Choi et al., 2016; Norman et al., 2016; Nycz et al., 2016; Yun S.-S. et al., 2017; Kim and Park, 2018; Lemerle et al., 2018; Burns et al., 2019). Yi et al. (2018) combined a linear SPA with Bowden cables to drive the flexion/extension of the fingers of a wearable hand exoskeleton.

The cables can be attached to a pulley or a gear when rotational motion is required at the output (Letier et al., 2006; Veneman et al., 2007; Vitiello et al., 2013; Agarwal et al., 2015; Cempini et al., 2015; Cappello et al., 2016; Choi et al., 2016; Dinh et al., 2016; Kalantari and Ghaffari, 2016; Thielbar et al., 2017; Yun S.-S. et al., 2017). Bidirectional rotational motion has often been achieved by connecting a pair of Bowden cables to a pulley (pull-pull torque transmission system). When linear motion is required at the output, the end of the cable most often serves as output and is attached directly to strategically chosen anchor points, e.g., on a glove or an exosuit either in unidirectional (Asbeck et al., 2015; Bartenbach et al., 2015; In et al., 2015; Nycz et al., 2015; Norman et al., 2016; Xiloyannis et al., 2016, 2019; Blumenschein et al., 2017; Kang et al., 2018; Kim and Park, 2018; Choi et al., 2019; Dwivedi et al., 2019; Rose and O'Malley, 2019; Tran et al., 2020) or in a push–pull configuration (Nycz et al., 2016; Randazzo et al., 2018).

### 2.2. Discussion of RAS Principles and Evaluation for Out-of-the-Lab Application

**Figure 1:**
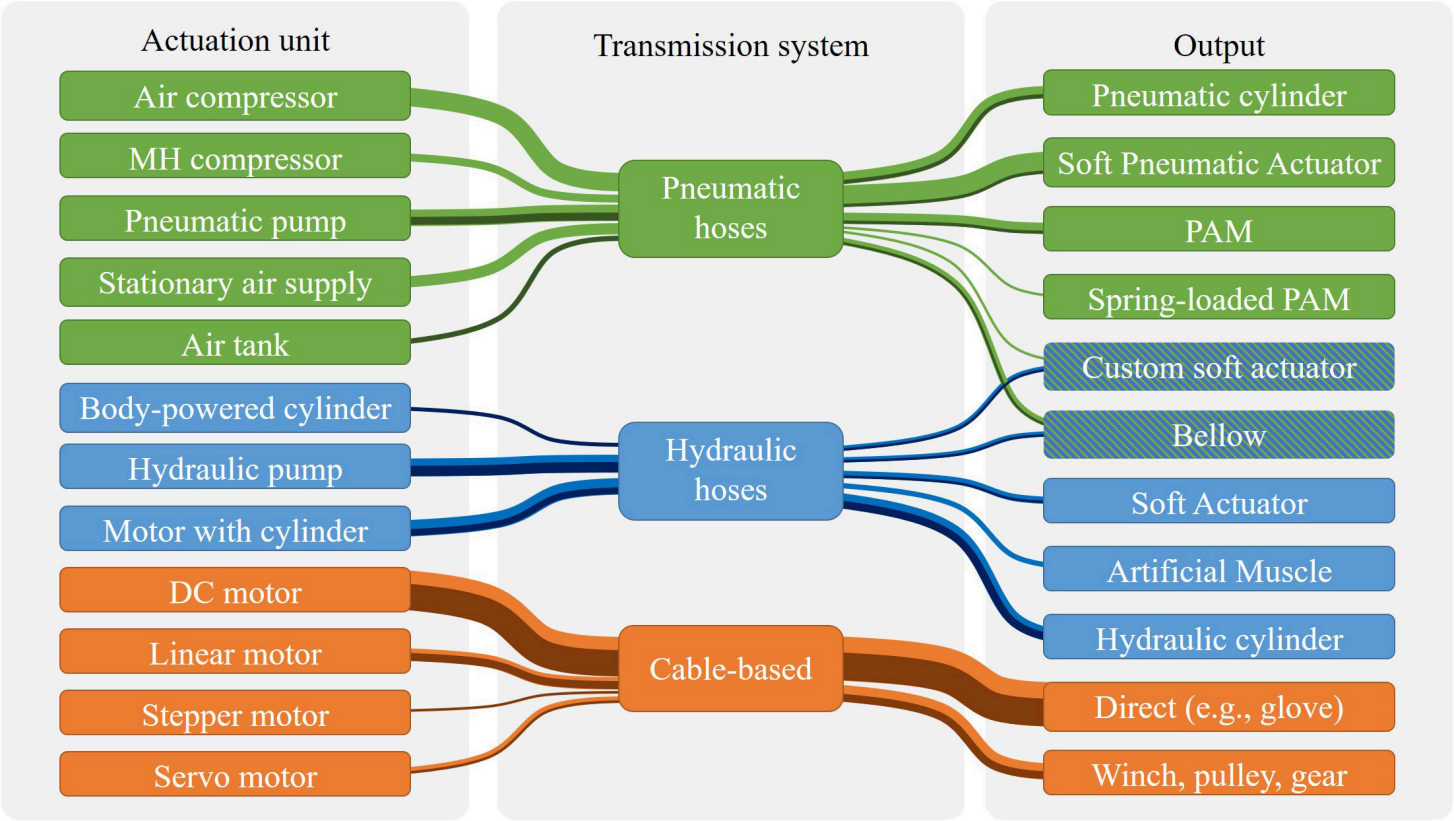
An overview of identified working principles of RAS from the literature research: Combinations of actuation units, transmission systems, and outputs are shown with line thickness indicating the estimated frequency of occurrence. Dark lines represent the combinations that can be considered fully wearable and thus potentially applicable in out-of-the-lab applications.

A summary of the RAS found in the literature is given in Figure 1, with each transmission system listed with the corresponding actuation units and outputs. Despite the intended application in fully wearable assistive robotics, only 25 out of the 81 (31%) reviewed RAS are fully wearable, assessed based on form factor, mass, and independence of permanently installed power sources. The most significant proportion (14 out of 25) of the identified fully wearable assistive devices are based on Bowden cables, mostly actuated by DC motors (10 out of 14) rather than, e.g., linear, stepper, or servo motors. In terms of the output of the RAS, fully wearable devices have been presented for almost all output principles. RAS that are not fully wearable and portable (e.g., tethered actuation units) were excluded from the evaluation for out-of-the-lab applications since these remain mandatory criteria for applications of wearable assistive devices. Consequently, stationary air supplies and compressors were not considered to be possible solutions due to the lack of lightweight and small pressure generators. MH compressors are more lightweight, and the chemical reaction is silent. However, pressure generation is slow and energy inefficient, and no fully wearable device incorporating MH has been presented.

The out-of-the-lab application of wearable assistive devices entails specific design requirements and challenges regarding RAS in addition to full wearability. The main arising requirements are high durability and low maintenance to enable continuous, longitudinal testing and application in clinics or user's homes where technical support is not readily available. In this regard, *maintenance* covers both daily handling by users (e.g., replacement and recharging of power source, donning/doffing, setup), and managing technical issues due to, e.g., wear and tear of the RAS. Durability is rarely reported in the literature and hard to rate for existing RAS. Here, we chose *efficiency* as a key requirement representing an indirect measure for the durability of a RAS since it strongly affects the wear on the mechanical transmission system. High overall efficiency is further a critical property of RAS regarding maximum output power, power consumption and with that battery runtime, and component selection, allowing the use of more compact and lightweight actuators and power sources to reduce the mass and volume of the overall RAS. However, the durability of a RAS needs to be specifically evaluated. To factor in the level of wearability of the RAS, *power density* (mechanical power per mass or volume) was chosen to summarize how compact and lightweight a RAS can be designed. Target values for *efficiency* and *power density* strongly depend on the application scenario but need to be maximized. *Safety* and *ergonomics* in terms of wearing comfort (soft design, i.e., not impeding the motion of the user), low noise emission, and dust- and waterproof design (e.g., against spillage in daily tasks or for disinfecting after clinical applications) gain additional importance.

To synthesize the findings of the literature review and evaluate the most suitable fully wearable RAS for out-of-the-lab applications, we rated the fully wearable combinations of actuation units, transmission systems, and outputs based on a Pugh analysis (Pugh, 1981) as shown in Table 1. The RAS concepts were compared and scored by weighting advantages against disadvantages for each solution and each requirement defined for out-of-the-lab applications (*maintenance, efficiency, power density, safety*, and *ergonomics*). Actuation units for cable-based approaches were categorized into linear motors with push-pull cables and rotary motors (DC, servo, and stepper) with pull–pull cables. The scores range from – – (worst) to + + (best), 0 indicating a “neutral” rating towards the other solutions. The final scores were established based on the ratings from 3 of the authors (JD, UATH, GS), which were based on objective information extracted from the literature reviews (see Supplementary Table 1) as well as their own experience. Great care was taken to select design requirements and their corresponding weightings neutrally. Nevertheless, it should be noted that when designing a RAS for a specific application scenario, additional requirements might need to be considered, or weightings might need to be adjusted.

**Table 1:** Pugh analysis of available fully wearable RAS concepts categorized by transmission system for out-of-the-lab applications.

**Subunits**	**Transmission**	**Pneumatic**				**Hydraulic**						**Cable-based**				**Weighting**

	**Actuation**	**Tank**	**Pump**			**Pump**			**Motor and cylinder**		**Body-** **power**	**Linear** **motor**		**Rotary** **motor**		
	**Output**	**PAM**	**Bellow**	**Cylinder**	**SPA**	**Bellow**	**Cylinder**	**SHA**	**HAM**	**Cylinder**	**Cylinder**	**Winch**	**Direct**	**Winch**	**Direct**	
Requirement	Maintenance	– –	0	+	0	– –	–	– –	– –	–	0	+	+	+	+	*2*
	Efficiency	–	0	–	0	+	0	+	+	0	0	–	0	0	+	*3*
	Power density	– –	–	–	0	+	+ +	+ +	0	+	+	+	+	0	0	*1*
	Safety	0	+	+	0	0	–	–	- -	–	+	+	+	+	+	*2*
	Ergonomics	– –	0	– –	0	+	0	+	+	–	0	+	0	+ +	+	*3*
	*Weighted score*	*−15*	*1*	*0*	*1*	*3*	*−2*	*2*	*−2*	*−6*	*3*	*5*	*5*	*10*	*10*	

*The weighting and weighted score for the design case with an assistive hand exoskeleton (section 2.3) is shown in italics in the last column and last row, respectively. Each + was counted as 1, and each – was counted as −1*.

#### 2.2.1. Maintenance

Wearable assistive devices should be easy to handle independently by end-users with sensorimotor impairment or by caregivers (e.g., therapists, family members, friends), including exchanging power sources, setting up (e.g., donning and doffing), or adjusting the device to individual users. Air tanks used to power pneumatic RAS feature limited capacity for small-scale designs, and the need to be refilled from air supplies often not accessible outside the lab (Luo et al., 2015). RAS powered by batteries are easier to handle by the users in terms of recharging, but also replacement since batteries can be considered more common in daily use. Body-powered systems do not require these steps at all, but they might require the user to readjust the transmission system's length or the anchor point, which might be challenging for users with sensorimotor impairment. Cable-based RAS can be designed to be separable from the actuated assistive device by, e.g., detaching the cables at anchor points, which is generally not possible for soft pneumatic and hydraulic actuators, bellows, or artificial muscles that are directly placed on assisted body parts. Depending on the specific design, this influences the ease of donning and doffing. The possibility to don and doff a cable-based RAS and the remotely actuated part of the device (e.g., glove, exosuit) separately might facilitate the setup. However, an additional step to connect the output of the RAS to the assistive device is required. Regarding technical maintenance, this modularity of cable-based RAS is beneficial, allowing quick repair or replacement of specific components of the RAS since structural failures are very common along the transmission path, e.g., especially breaking of the cable (Jeong et al., 2020). The increased wear and tear of soft pneumatic and hydraulic actuators, artificial muscles, bellows, and cylinders leads to an increased need for replacement compared to cable-based RAS. Further, leakage in hydraulic RAS can result in an increased need for service to refill hydraulic systems, depending on the specific design (e.g., after 80,000 grasp cycles in Smit et al., 2014b).

#### 2.2.2. Efficiency

High efficiency of the RAS influences several other properties of wearable robotic devices such as durability, power consumption, or maximum output power. Starting from the transmission, the mechanical transmission efficiency of push-and-pull mechanisms is usually around 70–80 % (Grosu et al., 2018), but can reach values of up to 96 % (Grosu et al., 2019). However, the mechanical transmission efficiency of cable-based systems highly depends on the bending angle (Agarwal et al., 2015; Nycz et al., 2016; Jeong and Cho, 2020). In terms of their actuation, DC motors reach high efficiencies. Depending on the output mechanism in pull–pull cable transmissions, friction losses in the additional structure (e.g., rack-and-pinion mechanisms) need to be considered. The efficiency of hydraulic and pneumatic actuators is one of their major limitations (Veale and Xie, 2016). The overall efficiency of pneumatic systems is often low since air is released into the surrounding (<30 %, Veale and Xie, 2016), but independent of the bending angle within the range that can be expected in wearable applications (0–180°). RAS based on pressure tanks require regulators leading to energy losses during the expansion (Noda et al., 2014; Park et al., 2014b). For hydraulic RAS, the transmission efficiency usually highly depends on the output type. At high flow speed, e.g., induced by small hose diameters, the hydraulic transmission efficiency decreases and becomes bending angle and transmission length dependent. Cylinders generally feature lower efficiency than bellows, artificial muscles, or soft pneumatic and hydraulic actuators due to friction losses in sealings. Overall, efficiency is a key criterion that needs to be considered and addressed during the design process of all RAS types to achieve high output forces and controllability.

#### 2.2.3. Power Density

In daily use of wearable assistive devices, wearers require the entire device to be lightweight to move around freely and profit from its assistance over several hours without causing discomfort or fatigue. Furthermore, the wearable assistive device should be compact and low profile to allow wearing clothing over it when going outside (Boser et al., 2018). High power density is required to reduce the mass and the volume of the RAS. In general, hydraulic transmission systems provide the highest power density out of the three RAS principles (Veale and Xie, 2016), especially compared to pneumatic RAS typically operated at lower pressures. However, actuation unit and output usually account for a larger proportion of the mass and volume of RAS than the transmission. Regarding hydraulic actuation units, hydraulic cylinders and linear motors have limited stroke length or result in bulky setups compared to more compact pumps. In contrast, cylinders can be operated at higher pressures allowing for smaller outputs (Smit and Plettenburg, 2011; Smit et al., 2014b). Similarly, air tanks storing liquid gas to power pneumatic RAS are more space consuming than pumps but can supply higher pressures. In terms of hydraulic and pneumatic outputs, PAM feature high power at low mass but have to be combined with springs or configured in pairs to provide bidirectional motion resulting in bulky outputs. Cylinder actuators can provide bidirectional motion at high power density in thin-walled designs (Plettenburg, 2005). Soft pneumatic and hydraulic actuators, and bellows are generally also more lightweight than PAM (Hong et al., 2020). For cable-based RAS, a push-pull configuration requires cables of larger diameter (and stiffness) than in a pull–pull configuration to transmit pushing forces. However, transmissions in a pull–pull setting require additional structures (e.g., second transmission cable and pulley at the output) to transform the unidirectional into bidirectional motion, increasing overall mass and volume. Electric motors can provide high strength at compact and lightweight design (Grosu et al., 2018). The power density of body-powered systems depends on the physical condition of the user, strongly varying among people with sensorimotor impairments. However, additional structures required for actuated assistive devices such as actuators or power sources are not required for body-powered devices, considerably reducing mass and volume.

#### 2.2.4. Safety

Safety aspects of a wearable assistive device address user interaction (e.g., exertion of forces and torques on the body) as well as potential risks for the user in case of device malfunction. Safe user interaction and safety mechanisms can be implemented computationally in RAS that feature high controllability (e.g., biomimetic trajectory control to avoid potentially hazardous motion induced on user). However, inherent compliance is considered to provide a higher level of safety and robustness (Veale and Xie, 2016). Therefore, the inherent compliance of pressurized air makes pneumatics a safe solution for user interaction in low-pressure regimes. Cable-based RAS also provide some compliance but are less controllable due to friction and backlash issues. Hydraulic RAS feature good controllability, smooth actuation, and high bandwidth (Veale and Xie, 2016), especially for bellow outputs that offer an almost constant force during extension. Soft pneumatic and hydraulic actuators and artificial muscles suffer from nonlinearities, reducing the controllability, and buckling, potentially causing harmful motion for the user (Martinez et al., 2008). Cylinders feature high linearity due to the constant cross-section along the whole stroke length but are affected by nonlinear friction making the control challenging (Huang and Chen, 2013). Body-powered RAS can be considered safe since the user has direct control over the device. Regarding RAS failures, hoses of pneumatic and hydraulic transmissions might burst under high pressure with potentially hazardous consequences, especially in RAS operated at high pressures (e.g., hydraulic cylinders). Failure of cable-based RAS due to break of transmission cables are a frequent malfunction, but solely results in de-powering the wearable assistive device, generally not harming the user.

#### 2.2.5. Ergonomics

In terms of ergonomics, high user acceptance for long-term, out-of-the-lab application depends on different criteria. High wearing comfort is required to prevent pressure points and discomfort over an extended usage period (up to 1 day in daily use) or hindering the user's motion. Accordingly, the weight of RAS needs to be considered. Therefore, the rating of the power density was factored in as an aspect of ergonomics. The RAS needs to be water- and dustproof, especially if the assistive device supports hand function during ADLs where the user gets in contact with liquid substances such as cleaning or grooming. Additionally, the RAS should be cleanable and, mainly in clinical applications, disinfectable. Noise emission should be kept to a minimum due to its disturbance and annoyance, e.g., during conversations or in public. Cable-based RAS, especially in pull–pull configuration realizable with thin cables, offer a soft and slender transmission (Jeong and Cho, 2020), increasing the wearing comfort. Hydraulic and pneumatic hoses tend to be more rigid and stiffen strongly when operated at high pressures, hindering the user's motion. Body-powered systems are often limited regarding wearing comfort (i.e., of the harness) and might lead to fatigue of the user over time (Biddiss et al., 2007). Regarding outputs, artificial muscles, soft pneumatic and hydraulic actuators, and bellows feature increased wearing comfort compared to cylinder-type outputs due to the often low-profile and biomimetic design allowing to fit the user's body shape and motion naturally (Yun Y. et al., 2017; Cappello et al., 2018; Hong et al., 2020). Hydraulic systems can be well controlled in a feed-forward manner, which allows for waterproof outputs free from electronics. However, even small leakage of fluids might cause inconveniences in daily life (e.g., spilling over clothes or food). Pneumatic systems have high noise emissions, e.g., in tank-based systems due to pressure control via valves and air release into the surrounding. Hydraulic and cable-based RAS operate more silently, primarily if actuated by electric motors. The noise emission of body-powered systems is very low.

### 2.3. Design Case: Fully Wearable Hand Exoskeleton

**Figure 2:**
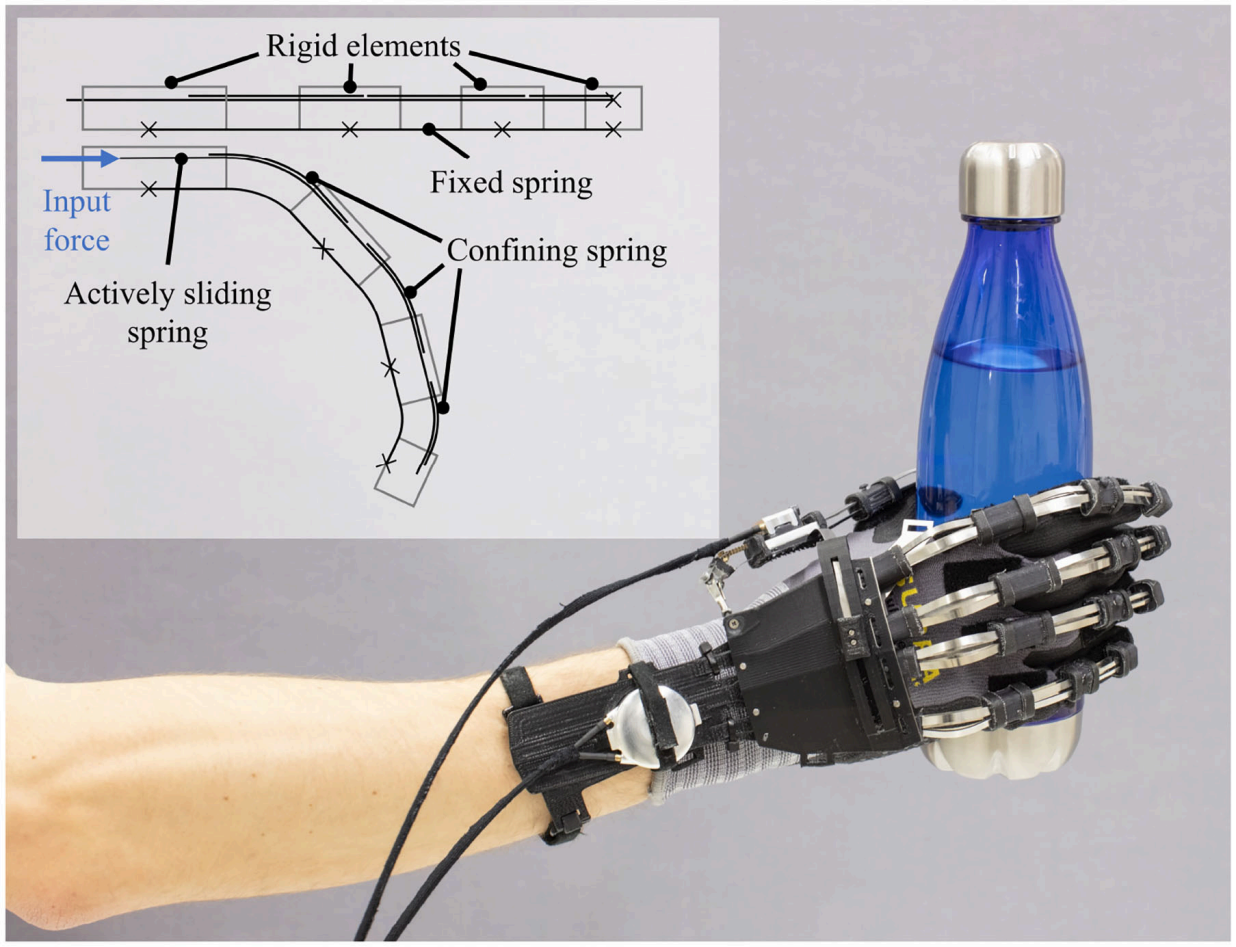
Assistive hand exoskeleton selected for the design case: The hand exoskeleton actively supports the flexion and extension of the four fingers and the thumb actuated by two separate RAS, as well as manual thumb opposition through a slider. The functionality is based on the illustrated three-layered spring mechanism. By linearly displacing a sliding spring mounted on top of a fixed spring, a bending motion of the springs is induced through the relative length change. Rigid elements and a third layer of spring blades connecting the springs confine the bending in three segments, resulting in biomimetic motion.

Building on the findings of the literature review, we selected, designed, and optimized a RAS in the context of the out-of-the-lab application of a fully wearable assistive device. As a design case, we chose the clinical and at-home application of the remote actuation unit of an assistive hand exoskeleton for adults and children with hand sensorimotor impairment presented in Bützer et al. (2019), Bützer et al. (2020) (Figure 2). The hand exoskeleton actively supports the four fingers' flexion and extension (index, middle, ring, and little finger) combined and the thumb separately. By additionally allowing thumb opposition through a passive slider (presented in detail in Bützer et al., 2020), the hand exoskeleton can assist users in performing the most relevant grasp types for daily life (e.g., power grasp, precision pinch, and lateral grasp). For the actuation of the hand exoskeleton, two separate RAS are required to provide a bidirectional, linear output to drive the three-layered spring mechanism implemented in the thumb and the fingers (Bützer et al., 2020) (illustrated in Figure 2). By linearly displacing a sliding spring blade mounted on top of a fixed spring blade, a bending motion of the springs is induced through the relative length change. Rigid elements and a third layer of spring blades connecting the springs confine the bending in three segments, resulting in a biomimetic motion similar to flexion/extension of a human finger.

Since the hand exoskeleton proved feasible in first user studies, longitudinal testing in daily life and clinical application is targeted to gather additional insights into usability and applicability in out-of-the-lab applications. In Nycz et al. (2016) and Hofmann et al. (2018), cable-based RAS for previous versions of the hand exoskeleton have been presented, of which the latter has been used in user tests conducted so far. However, moving toward application in highly intensive, task-oriented therapy, and daily use, the previous RAS performed poorly in durability. Due to the increased usage time and grasping frequency, malfunctions of the RAS occurred after few hours of use (<2,000 grasp cycles). Furthermore, the previous RAS struggled with low transmission efficiency and insufficient output force. To make the hand exoskeleton ready for long-term use in daily life in an at-home environment and the integration in clinics, we developed and optimized a RAS for these out-of-the-lab applications.

#### 2.3.1. Quantitative Design Requirements and Selection of RAS Principle

To identify the optimal RAS principle for this specific design case, we weighted the criteria rated in the Pugh analysis regarding their importance from 1 (low importance) to 3 (critical). First and foremost, *efficiency* was rated critical (3 on importance scale) since it strongly influences the durability but also the need for maintenance of the RAS. Transmission efficiency of 90 % in the operating range relevant for the application scenario allows for a sufficiently accurate force control without taking the bending angle along the transmission path into account. This allows to simplify not only the output force control but also to reduce the mass and the volume and increase the durability and robustness of the RAS. In the application of the hand exoskeleton, bending angles of 135 ± 45° can be expected in daily grasping tasks, assuming the cable to be routed along the arm from the back to the hand (Desmurget et al., 1995; Butler et al., 2010). *Ergonomics* and appearance are further essential requirements (3) that are decisive for device acceptance by users (Boser et al., 2018). In that sense, the entire RAS is required to be water- and dustproof, nonobstructive to not restrict the user's movements, and easy to handle by a user with unilateral or bilateral arm/hand impairment. Further, *safety* is an important criterion when interfacing with users (2), but difficult to be evaluated and guaranteed. Low *maintenance* was considered important (2). In daily life, end-users of hand exoskeletons are often accompanied by caregivers, yet not by technical staff. Therefore, basic operations such as exchanging a battery can be considered feasible, but the need for technical maintenance such as exchanging broken components should be avoided, calling for a highly durable RAS. *Power density* was given a lower importance rating (1), although still very relevant to design lightweight RAS. The entire RAS needs to be fully wearable, e.g., in form of a compact and lightweight backpack to allow the users to move around freely. According to Moore et al. (2007) and Hong et al. (2008), 10–15 % of body weight depicts the cutoff point for the acceptable mass of backpacks for children. For 6-year-old children who have cerebral palsy, representing the youngest potential end-users of the hand exoskeleton, the maximum backpack mass would correspond to 1.25 kg (Krick et al., 1996). In terms of specific mechanical requirements for the application with the selected hand exoskeleton, the RAS needs to provide a force between 50 and 60 N per finger and a linear stroke of 40 mm to achieve full flexion of the fingers and fingertip forces relevant for daily grasping tasks (i.e., 5 N, Bützer et al., 2019). To perform ADLs, hand opening and closing at 0.5 Hz is desired. Since the four fingers are actuated simultaneously in the selected hand exoskeleton, the RAS needs to provide at least 200 N force at 20 mm s^−1^ velocity at the output.

Based on the weighting of the design requirements, a total weighted score for each RAS principle was calculated. From the rating presented in italics in Table 1, a cable-based pull–pull transmission was identified as the most suitable RAS principle for out-of-the-lab applications in hand exoskeletons to provide grasp assistance in daily life and therapy. Therefore, this evaluation supports the previous RAS selection made for the hand exoskeleton in Hofmann et al. (2018).

**Figure 3:**
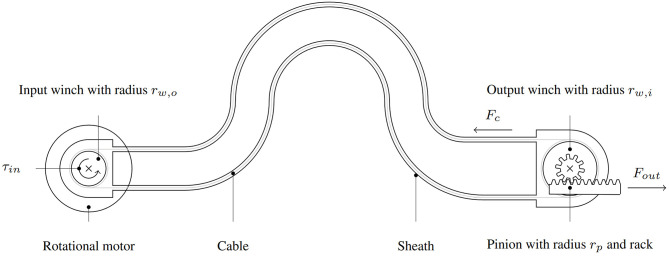
Working principle of the proposed Bowden-cable-based RAS: The transmission system consists of traction cables that are connected to an input winch and an output winch. A rotary electrical motor applies a torque τ_*in*_ to the input winch converting it to a cable tension *F*_*c*_. At the output, a rack-and-pinion mechanism converts the torque from the output winch into a force *F*_*out*_.

Since a bidirectional, linear output is required to actuate the hand exoskeleton, we propose a RAS based on a pull–pull Bowden cable transmission similar to Hofmann et al. (2018). The proposed RAS consists of an input winch at the actuation unit and a rack-and-pinion mechanism that converts rotation to linear motion and torque to force at the output (Figure 3). A rotational motor drives the input winch. This configuration features high scalability compared to direct transmissions at the output, allowing the use of the same RAS principle for the actuation of the fingers and the thumb by adapting the output mechanism only.

#### 2.3.2. Component Selection and Implementation of a Cable-Based RAS

We designed a cable-based RAS to actuate the four long fingers of the hand exoskeleton. By increasing the efficiency, we aimed to develop a more durable, compact, and lightweight RAS. The RAS presented in Hofmann et al. (2018) relied on sensors to accurately control the output force due to the bending angle-dependent friction in the transmission, adding mass, volume, and complexity (e.g., sensors, additional electronics, and cables). To avoid the need for these additional structures and allow the use of lighter, small-scale actuators, transmission components, and power sources, the bending angle-dependent power losses along the transmission need to be minimized (Popov et al., 2017). Selecting axially stiff (against compression due to cable traction) sheaths with a longitudinal construction of flat-band steel rather than spiral-spring type constructions (Letier et al., 2006; Chen D. et al., 2014) and coated cables can reduce the friction losses and wear (Xiloyannis et al., 2016; Jeong et al., 2020). Stiff sheaths further prevent bending of the cable transmission at very small deflection radii, which would increase the wear on the cable and, consequently, decrease the transmission efficiency over time (Letier et al., 2006). Accordingly, custom-made, flat-band steel sheaths (inner diameter 0.6 mm, outer diameter 1.1 mm) were used due to their small diameter, high flexibility toward bending but high axial stiffness, and beneficial friction properties. High transmission efficiency and increased durability can further be achieved by evaluating optimal material pairings of sheaths and cables (Letier et al., 2006). Two different cable types fitting into the sheaths were included for further evaluation. Custom-made, Teflon-coated steel cables (diameter 0.3 mm) were selected as a trade-off between cable stiffness and transmittable force (70 N). Micro-coated PE fiber wires (Spiderwire stealth smooth 8, Columbia, USA; diameter 0.39 mm) were additionally evaluated, as they feature greater bearing capacity (180 N) and higher flexibility at comparable diameter to the steel cables. In a next mechanical design step, the radius of the output winch *r*_*w,o*_ was calculated as a function of the required output force *F*_*out*_ based on maximum cable tension *F*_*c,max*_, the expected efficiency of the transmission system η_*t*_ at the maximally expected bending angle (90 % at 180°) as well as the expected efficiency of the rack and pinion η_*rp*_ (95 %):

(1) $$\begin{array}{r} r_{w,o}\geq \frac{S\cdot F_{out}\cdot r_{p}}{F_{c,max}\cdot \eta_{t}\cdot \eta_{rp}} \end{array}$$

where S is the safety factor (here set to 1.5) and *r*_*p*_ is the pitch radius of pinion.

The rack was scaled proportionally in width and height for sufficient output force *F*_*out*_ and in length for the required stroke. Decreasing cable speed was shown to increase the efficiency of RAS (Chen D. et al., 2014). To achieve the desired output force and to keep the required cable speed low, we set the gear ratio of the output mechanism to 5 (*r*_*w,o*_ = 12.5 mm, *r*_*p*_ = 2.5 mm). The motor and the input winch connected to it were selected according to the required cable speed and tension at the actuation unit. The output force *F*_*out*_ at the rack relates to the torque at the input winch τ_*in*_ according to:

(2) $$\begin{array}{r} F_{out}=\frac{\exp\left(-\mu \cdot \sigma \right)\cdot r_{w,o}\cdot \eta_{rp}}{r_{w,i}\cdot r_{p}}\cdot \tau_{in} \end{array}$$

where μ is the coefficient of friction in the Bowden cable,
σ is the bending angle of the Bowden cable,
*r*_*w,o*_ is the radius of output winch, and
*r*_*w,i*_ is the radius of input winch.

The required input torque τ_*in*_ depends on *r*_*w,i*_ (Equation 2). It can be achieved with an arbitrary number of motor/winch combinations. However, *r*_*w,i*_ must be large enough to meet the desired maximal output speed. We chose a rather small radius for the input winch (*r*_*w,i*_ = 8 mm) to increase the force-to-velocity ratio of the transmission cable. Accordingly, a motor delivering a torque of at least 0.337 N m at a speed of up to 119 rpm should be chosen. DC motors are often recommended and the most used actuators for fully wearable cable-based RAS due to their quiet operation and lightweight yet powerful design (Popov et al., 2017). We selected a DC motor (DCX22S, Maxon Motor, Switzerland) combined with a planetary gear with a reduction ratio of 44:1 (GPX22; Maxon Motor) for its high power density and high efficiency (85.2% for the DC motor, 81% for the gear). A compact motor driver (ESCON Module 24/2; Maxon Motor) was chosen to control the motor current and torque according to a pulse width modulation (PWM) signal provided by a microcontroller (Arduino Yun Mini, Arduino, Italy).

#### 2.3.3. Evaluation Methods and Outcome Measures

**Figure 4:**
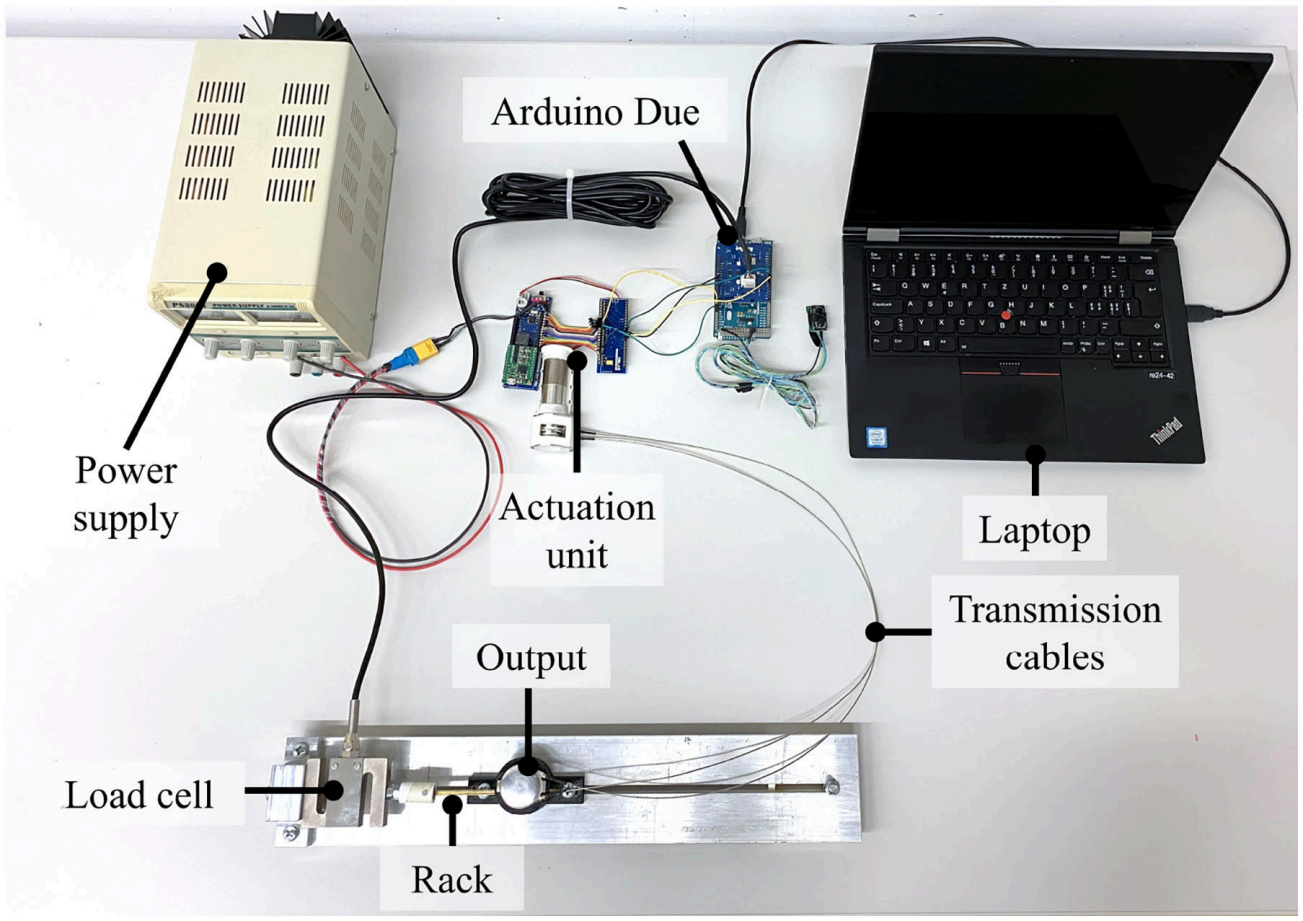
Test bench for RAS evaluation: A load cell, directly attached to the rack at the output of the proposed RAS, measured the output force and behavior. An Arduino Due microcontroller and laptop were used to provide the control signal to the actuation unit and record the load cell measurements. A stationary power supply powered the RAS. The test bench was adapted by interposing a tension spring between the load cell and the rack for the power characterization of the RAS, and connecting the hand exoskeleton instead of the load cell at the output for the durability evaluation.

The proposed RAS was evaluated with respect to the specific design requirements for out-of-the-lab applications. First, maximum output force, efficiency, durability, and power density (i.e., power, mass, and volume) were determined quantitatively and optimized in bench tests (Figure 4). Further aspects such as ergonomic requirements (e.g., wearing comfort) were evaluated based on subjective feedback collected in user studies and qualitatively from observations.

##### 2.3.3.1. Test bench evaluation

To investigate and optimize the output behavior of the proposed RAS in terms of maximum output force, efficiency, and durability, different reference profiles of the input current were applied as a control input to the motor drivers. We characterized the output behavior of the RAS to determine the control input that minimizes force peaks in the cables that would potentially decrease their durability. Standard input profiles (step, ramp, quadratic, and cubic parabolic) were evaluated. Additionally, a fifth-order polynomial minimum jerk function was investigated. The minimum jerk function represents the trajectory that natural human upper-limb movements tend to follow (Flash and Hogan, 1985), minimizing wear and tear on the human biomechanical system (Yazdani et al., 2012).

Time-dependent input profiles were applied over a rise time of 1 s, corresponding to a hand opening and closing frequency of 0.5 Hz. The motor current was increased step-wise until the maximum duty cycle was reached (motor currents of 0.39 to 1.46 A in steps of 0.12 A) or the cable tore. The output force at the rack of the RAS was measured using a load cell (Advanced Force Gauge 100 N; Mecmesin, UK). In the test bench (Figure 4), an Arduino Due microcontroller (Arduino, Italy) was used to provide the PWM signal to the motor controller and record the load cell measurement. A stationary power supply was used to power the RAS. The tests were conducted with both the steel and PE wires to identify the most favorable material pairing of cables and sheath. The optimal control input and cables regarding output behavior (i.e., achievable output force, least wear on the system in terms of force peaks) were retained for further evaluation.

The maximum output force, transmission efficiency, and the influence of the bending angle were investigated by measuring the output force using the force gauge for different input torques, as described above, and bending angles (0–180° in steps of 30°, at 270°, and 360°).

The output power *P* of the system was measured with a tension spring (k = 3.023 N mm^−1^; Durovis, Luxembourg), which connected the load cell to the rack at the output. The properties of the spring were chosen such that it emulated the mechanical properties of the hand exoskeleton. In this setup, the RAS was used to increase the tension on the spring from a force *F*_1_ to *F*_2_ > *F*_1_ by increasing the input current from 0.39 A to 1.1 A in an open-loop manner over a time span Δ*t*:

(3) $$\begin{array}{r} P=\frac{{F_{2}}^{2}-{F_{1}}^{2}}{2\cdot \Delta t\cdot k}. \end{array}$$

The mass and the volume of each part of the RAS, including the actuation unit, transmission system, and output, were measured. For an objective comparison to other systems, relative values were used for the power density, as recommended in Moreno et al. (2008). The power-to-mass ratio *P*_*m*_ and power-to-volume ratio *P*_*v*_ is thereby defined as:

(4) $$\begin{array}{r} P_{m}=P/m;P_{v}=P/V \end{array}$$

where *m* is the mass and *V* is the volume.

Finally, the durability of the RAS was evaluated by connecting the hand exoskeleton to the output and measuring the number of grasp cycles (one opening and closing) until component failure. The transmission cable was bent by an angle of 135° to mimic the application scenario. The bending radius was chosen arbitrarily (approximately 15 cm) since it has a negligible effect on the performance of the Bowden cables (Chen L. et al., 2014). The motor current was chosen based on the evaluation of the transmission efficiency so that the targeted 200 N output force was reached. Failure modes were recorded to identify the three components of the RAS that break the earliest, representing the components that require the most frequent maintenance and need to be optimized. Broken components from the first two failure modes were replaced before continuing the evaluation. The test was concluded after the failure of the third component.

##### 2.3.3.2. User evaluation

To gather qualitative feedback on ergonomic aspects, the proposed RAS was applied in end-user tests with two SCI subjects and 11 children with hand sensorimotor impairment in an out-of-the-lab, clinical environment. The subjects used the hand exoskeleton actuated by the proposed RAS for at least 1 h while performing grasping tasks relevant for ADLs in task-oriented training or free testing (e.g., pouring and drinking water from a bottle). The subjects were asked to rate the wearing comfort of the entire hand exoskeleton system (i.e., RAS and hand exoskeleton) on a Likert scale from 1 (not comfortable at all) to 5 (very comfortable) and provide feedback in open, unstructured discussion. The experimental procedures were approved by the ethics committee of ETH Zurich (2018-N-90) and the ethics committee of the Canton of Zurich (KEK-2019-00409). All children and their legal guardians provided verbal consent to participate in the study: parents, adolescents aged 14 years and older, and the adult SCI subjects provided written informed consent.

## 3. Results

### 3.1. Final Prototype of the RAS

**Figure 5:**
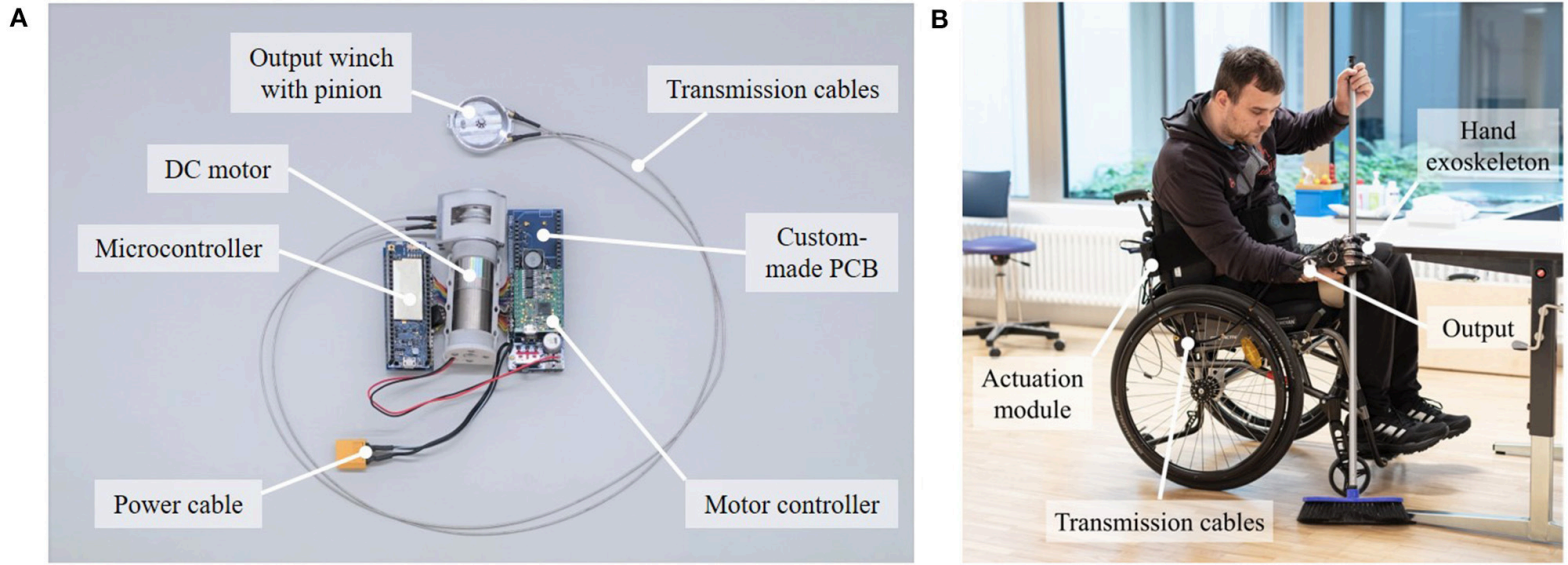
Overview and use case of the developed cable-based RAS: **(A)** A DC motor actuates the pull–pull cable transmission system. At the output, rotational motion and torque are translated into linear motion and force via a rack-and-pinion mechanism. A microcontroller and motor controller, placed on custom-made printed circuit boards, control the motor current. **(B)** An SCI subject wears the hand exoskeleton actuated by the developed RAS to firmly grasp a broom. The fully wearable RAS integrated into the actuation module is mounted on the backrest of the wheelchair. The flexible transmission allows the user to move the arm freely.

**Figure 6:**
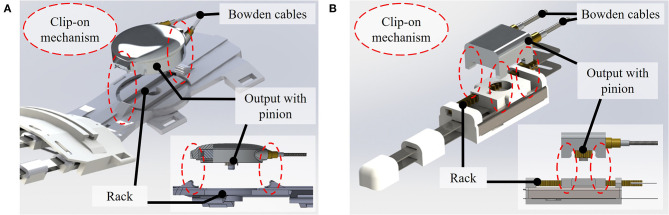
Output of the RAS for the actuation of **(A)** the fingers and **(B)** the thumb: A clip-on mechanism, highlighted in dashed red circles, allows to attach and detach the output of the RAS from the hand exoskeleton.

The developed RAS is shown in Figure 5A. For the final version, the PE wires were selected as the transmission cable based on the test bench evaluation, as detailed in the following. The actuation unit consists of a DC motor combined with a planetary gear. The motor driver and microcontroller are placed on custom-made printed circuit boards (PCBs) carrying the power electronics. Flat grooved ball bearings (FL678ZZ; Misumi, Japan) and small-diameter grooved ball bearings (EZO 683; Sapporo Precision Inc., Japan) guide the custom-made input winch and output winch, respectively. At the output, the torque is transmitted to force from a pinion (10 Z M 0.5; Reely, Germany) to a 50 mm long rack (brass rack M 0.5; Reely, Germany) integrated into the hand exoskeleton (shown in detail in Supplementary Figure 3). The output can be attached to the hand exoskeleton through a clip-on mechanism (Figure 6). Thereby, donning/doffing and potentially required technical maintenance (e.g., replacement of broken cables) are facilitated since the RAS can be decoupled from the hand exoskeleton. The overall mass of the developed RAS is 259 g, with the actuation unit accounting for the most substantial proportion (211 g).

A RAS based on the same components was built to actuate the thumb of the hand exoskeleton by adapting the output only and scaling the output winch and rack-and-pinion mechanism, highlighting the scalability of the approach. The actuation units and electronics were integrated into a textile-based soft, sleek, and lightweight casing to ensure high wearing comfort, referred to as the actuation module. The actuation module is fully wearable as a backpack or can be mounted on the backrest of a wheelchair (Figure 5B). The transmission sheaths were wrapped with waterproof, medical kinesiotape (Kineasy; AcuMax Med AG, Switzerland) to protect them against environmental influences. The total mass of the actuation module, including the entire RAS (actuation unit, transmission system, and output) for the fingers and the thumb as well as the casing, is 560 g. Batteries of different sizes and capacities can be connected via the power cable. For instance, a LiPo battery weighting 137 g was used in Bützer et al. (2020) to reach a battery runtime of 1,200 grasp cycles, covering approximately 2 h of use.

### 3.2. RAS Evaluation

The main results of the evaluation of the developed RAS are presented in Table 2 and compared to previous RAS for the application with the hand exoskeleton (Nycz et al., 2016; Hofmann et al., 2018). The maximum output force, power, transmission efficiency, durability, power-to-mass ratio, and power-to-volume ratio were improved by factors of 1.53, 2.36, 1.38, 5.71, 5.0, and 6.19 with respect to the RAS presented in Hofmann et al. (2018), respectively.

**Table 2:** Performance metrics of the proposed optimized RAS and previous versions.

	**Optimized RAS**	**Hofmann et al. (2018)**	**Nycz et al. (2016)**
		**Performance**		
*F*_*max*_ [N]	230 ± 4	150 ± 5	28 ± 4
*P*_*max*_ [W]	2.6	1.1	0.1
η_*transmission*_ [%]	90	65	n/a
Durability [#grasp cycles]	> 20,000	<3,500	n/a
		**Mass [g]**		
Actuation unit	211	516	127
Transm. system	28	33	31
Output	20	20	5
Overall	259	569	163
		**Volume [cm**^**3**^**]**		
Actuation unit	246	682	77
Transm. system	8	6	6
Output	8	9	2
Overall	262	697	85
	**Power-to-mass ratio [W/kg]**		
Overall	10.0	2.0	0.9
	**Power-to-volume ratio [kW/m**^**3**^**]**		
Overall	9.9	1.6	1.8

*P_max_ – maximum mechanical output power, η_transmission_ – transmission efficiency for 135° bending condition*.

#### 3.2.1. Control Input Trajectory and Transmission Cable

**Figure 7:**
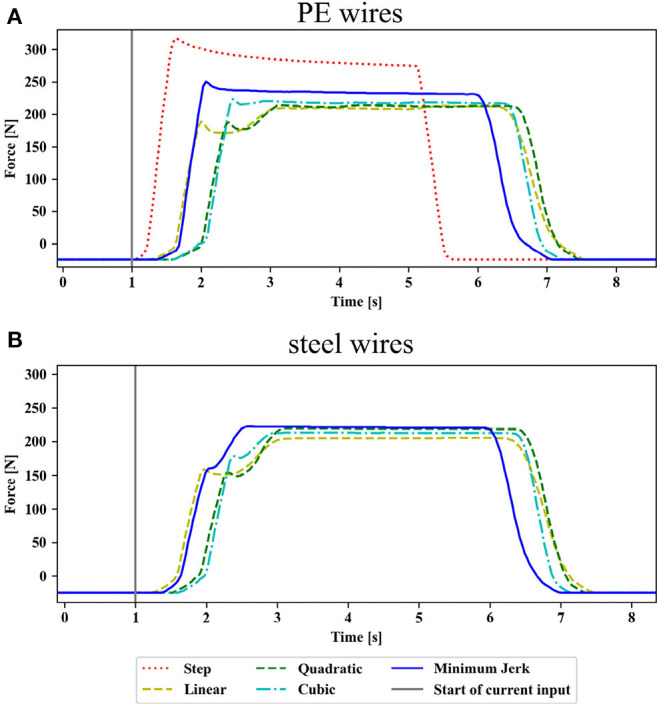
Comparison of the output force profiles for different control inputs using **(A)** polyethylene (PE) wires and **(B)** steel wires for transmission: The output profiles are aligned with respect to the starting time of the input trajectory (vertical gray line). The final current for all input trajectories was 1.46 A. All input trajectories lead to an initial force peak, which was higher for the PE wires. The output force drops for the linear, quadratic, and cubic input after the initial peak before rising again. The steady-state is reached the fastest for the minimum jerk input without rising output force after the initial peak. For all control inputs, the final force level was lower for the steel wires compared to the PE wires. The output force for a step input on the steel wires is not shown as the wire tore at lower motor currents than 1.46 A.

The steel and PE wires showed similar transmission behaviors for changing input trajectories. A step input resulted in the fastest rising and highest output forces of the input trajectories. However, the high force peaks led to cable breaks after a few repetitions before reaching the maximum motor current. The steel and PE wires sustained 3–4 and 6–8 repetitions of the step input, respectively. The output forces reached 209 N with peaks at 239 N, and 290 N with peaks at 320 N for the steel and PE wires, respectively. Among the time-dependent input trajectories, the minimum jerk input reached the highest force output of up to 229.8 ± 4.7 N compared to 217.3 ± 0.4, 214.0 ± 2.2, and 211.7 ± 1.2 N for the cubic, quadratic, and linear input, respectively, for the PE wires. All input trajectories led to an initial force peak. From Figure 7A, it can be observed that the output force dropped for the linear, quadratic, and cubic input after the initial peak before rising again. The steady-state was reached the fastest (after approximately 1.8 s) after input onset for the minimum jerk input without rising output force after the initial peak. However, the minimum jerk input did not result in a minimal jerk output trajectory. A similar behavior was observed for the steel cables but at slightly lower maximum force levels (Figure 7B). The output force reached 223 ± 5.2 N, 212.0 ± 1.2, 217.0 ± 1.7, and 204.1 ± 0.8 N for the minimum jerk, cubic, quadratic, and linear input, respectively. A comparison of the applied input current trajectories against the resulting output force profile is presented in the Supplementary Material for the PE wires (Supplementary Figure 1) and the steel wires (Supplementary Figure 2). Based on these results, the PE wires and a minimum jerk input were chosen for further evaluation.

#### 3.2.2. Maximum Output Force, Power, and Transmission Efficiency

**Figure 8:**
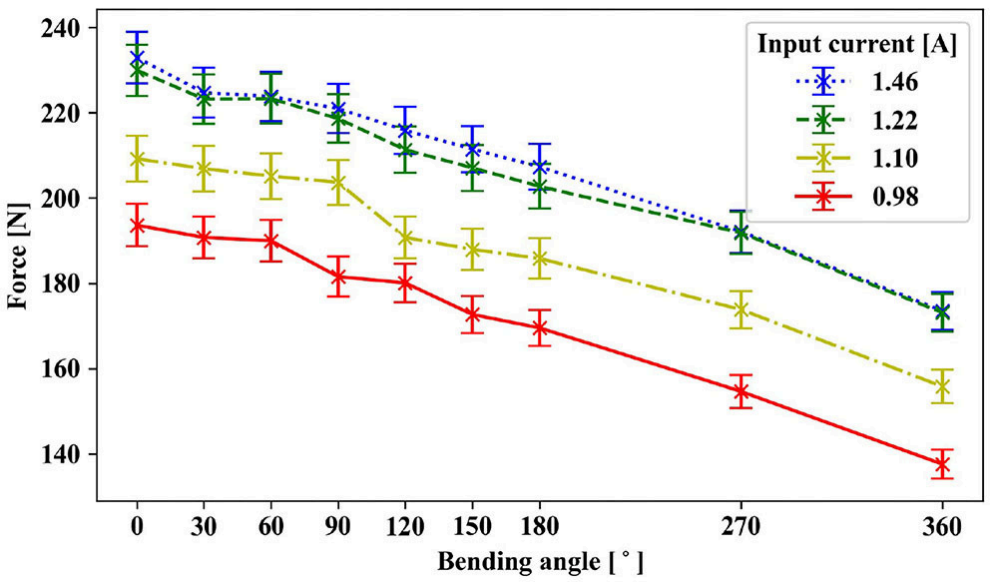
Output force for different bending angles and motor currents: The achievable output force decreases with increasing bending angle and decreasing motor current, corresponding to the applied motor torque at the input.

**Figure 9:**
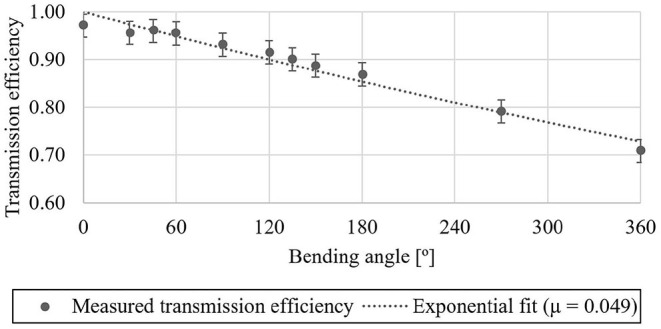
Force transmission efficiency as a function of the cable bending angle: The efficiency decreases exponentially with increasing bending angle according to the capstan equation (Equation 2). In the application scenario with a hand exoskeleton cable bending angles between 90 and 180° can be expected.

The maximum output force of the RAS decreased with increasing bending angle and decreasing motor current (Figure 8). Figure 9 shows the transmission efficiency at the evaluated bending angles and the capstan equation

(5) $$\begin{array}{r} F_{t,o}=F_{t,i}\cdot \exp\left(-\mu \cdot \sigma \right) \end{array}$$

fitted to the dataset. In the unbent condition, a maximum output force of 229.8 ± 4.7 N at a transmission efficiency of 97 % was reached for a maximum motor current of 1.46 A. The maximum output force and efficiency decreased to 207.3 ± 5.2 N and 87 % for a bending angle of 180° and to 173.6 ± 4.3 N and 71% for a bending angle of 360°, respectively. Factoring in the efficiency of the DC motor and gear, the overall efficiency of the RAS was 67 and 60 % for the unbent and 180° bent condition, respectively. In the typical operating range of 90–180° in the application with a hand exoskeleton, the required output force of 200 N was reached for motor currents above 1.22 A. For motor currents higher than 1.22 A, the output force did not increase substantially. The maximum output power of the RAS was 2.6 W, resulting in a overall power-to-mass ratio of 10.0 W kg^−1^ and a power-to-volume ratio of 9.9 kW m^−3^.

#### 3.2.3. Durability Evaluation

The RAS achieved a durability of over 20,000 grasp cycles before the first failure occurred. A PE wire was the first component to break close to the output winch after 21,991 grasp cycles. The wires were replaced before continuing the test. The couplers fixing the Bowden sheath loosened as the second failure mode, causing the sheath to be pulled into the actuation unit after 20,749 additional grasp cycles (component durability of 42,740 grasp cycles). The couplers and the transmission cables were replaced to avoid interruption of the evaluation before the failure of the next weakest component. Finally, the rack-and-pinion mechanism at the output broke after 12,546 further grasp cycles resulting in component-specific durability of approximately 55,000 grasp cycles. A summary of failure modes and component-specific durabilities is shown in Table 3.

**Table 3:** Summary of the durability evaluation to identify the three weakest components of the RAS.

**Number of grasp cycles** **until failure**	**Failure mode**	**Action before continuation** **of evaluation**
21,991	PE wire tore	All PE wires replaced
42,740	Bowden sheath coupler loosened	Couplers and all PE wires replaced
55,286	Rack-and-pinion mechanism broke	End of evaluation

#### 3.2.4. User Experience

The RAS as part of the hand exoskeleton system was perceived comfortable to wear, indicated by a mean rating of 4.0 ± 0.8 out of 5 on the Likert scale. Furthermore, it was observable that the cable-based transmission did not hinder the arm motion of the users. Most of the subjects mentioned in the open discussion that the entire hand exoskeleton, including the RAS, is non-obstructive and lightweight. One child (7 years old, muscular disease) mentioned the actuation module carried as a backpack becoming heavy toward the end of a 1-h therapy session with the hand exoskeleton. The RAS endured all user tests (approximately 10 h of use) without failure, and no adverse advents occurred in the user tests.

## 4. Discussion

In this paper, we presented the selection and optimization of a RAS for a fully wearable assistive device moving toward out-of-the-lab applications, such as clinical evaluation or longitudinal testing in user's homes, thereby aiming to overcome limitations of typical temporally short testing sessions of research prototypes performed in lab settings. We provided a literature review on RAS for wearable assistive devices and evaluated these in light of key requirements for out-of-the-lab applications, including durability, efficiency, ergonomics, and low maintenance to support the selection of a fully wearable RAS. Following this, an optimized fully wearable RAS for out-of-the-lab applications of an assistive hand exoskeleton was developed and evaluated. We identified cable-based transmission as the most suitable approach for the specific design case. The final RAS consists of pull–pull cable transmissions via PE wires in flat-band steel sheaths that transmit the power provided by a DC motor to a rack-and-pinion mechanism at the output.

The developed RAS features high durability of > 20,000 cycles before the first failure occurs, which allowed its application without technical issues for several hours in user studies in out-of-the-lab scenarios. While many research projects target the translation to out-of-the-lab applications (e.g., home use), the durability of RAS is rarely evaluated, allowing limited insight into applicability and usability of developed wearable assistive devices (Jeong et al., 2020). Often, durability is only reported for specific components, e.g., a battery runtime of 3.8 h powering a soft robotic glove in Polygerinos et al. (2015), or 5,000 cycles for fluidic fabric muscle sheets intended to be integrated as an output in wearable soft robotics in Zhu et al. (2020). The achieved durability for the presented entire RAS of > 20,000 cycles represents a benchmark for future durability evaluation of RAS for wearable assistive devices.

Despite the improved performance compared to previous versions, the durability of the presented RAS still needs to be further validated in daily, out-of-the-lab applications of the hand exoskeleton. In Bullock et al. (2013), it was shown that during intensive bimanual work (e.g., housekeeping), up to 600–700 grasps are performed per hour. In daily tasks performed by a person with sensorimotor impairment, the number of grasps per hour can be assumed to be much lower. Participants of the end-user tests mentioned that they would like to use the hand exoskeleton not throughout the whole day but in specific daily tasks that they cannot master without assistance (e.g., require additional grasp force). In first tests with a pediatric version of the hand exoskeleton, we recorded approximately 100 grasps per hour of therapy, including donning and doffing time. Accordingly, users of the hand exoskeleton can be expected to perform around 100–150 grasps per hour. Usage time of 7 h/day would result in approximately 1,000 grasps per day. Considering the determined durability of the RAS of slightly above 20,000 grasp cycles, around 20 days of use could be achieved until a technical failure of the RAS occurs. Optimally, the operation of the hand exoskeleton system should be guaranteed over several months or even years.

The presented RAS reached a transmission efficiency of above 86 % in the intended application scenario with cable bending angles ranging from 90 to 180° and, therefore, outperforms cable-based transmissions presented in the literature where efficiency was reported. Chen D. et al. (2014) and Jeong and Cho (2015) achieved 67 and 76 % transmission efficiency at 180° cable bending, respectively, resulting in the need for friction feed-forward control in their application scenario. Schmidt et al. (2017) presented a higher efficiency of 86 % for their tendon actuator, although not reporting the influence of the bending angle. The presented RAS reaches a transmission efficiency comparable to hydraulic transmissions with cylinders at the output, which are generally expected to be higher than in cable-based systems (Smit et al., 2014a) (e.g., 80 % for electro-hydraulic transmission in Bechet and Ohnishi, 2014). However, the proposed cable-based RAS does not suffer from ergonomic and maintenance-related issues present in hydraulic transmissions due to, e.g., small leakage, noise emission, or more complex control. Furthermore, the modularity and separability of the proposed cable-based RAS from the hand exoskeleton would allow for facilitated maintenance compared to previously presented pneumatic or hydraulic wearable assistive devices.

The high transmission efficiency for the presented RAS results from an in-depth optimization of components (e.g., material pairing) and design parameters (control input trajectories). The selected PE wires' diameter accurately matches the steel sheath size restricting the radial play of the cables or potential buckling. Accordingly, cables with a tighter fit in sheaths might be a beneficial choice for transmission efficiency. A minimum jerk trajectory was determined to increase the force throughput, reduce force peaks in the transmission, and potentially increase efficiency and durability of cable-based RAS. Minimizing jerk, the second time derivative of velocity, might reduce velocity-dependent friction phenomena (e.g., viscous friction). However, stiction remains an issue for smoothly rising input functions compared to fast-rising inputs (e.g., a step function) due to its nonlinear properties, leading to delays observable in the rising output force in Figure 7. Additionally, smooth trajectories are essential for safe human–robot interaction (Amirabdollahian et al., 2002). Because of the transmission properties, the output behavior of the presented RAS does not follow a minimum jerk trajectory. The transfer behavior to hand exoskeleton motion should be further analyzed to investigate whether a minimum jerk trajectory at the output results in a more comfortable motion for users than the current output trajectory. However, we speculate that a well-controlled output trajectory is less critical for the actuation of soft, compliant wearable assistive devices such as the hand exoskeleton selected as the design case.

Despite the high transmission efficiency, the overall efficiency of the proposed RAS, including the actuation unit, is not higher than 60 %. A main limiting factor of the overall efficiency is the need for a gear to meet the typically high torques at low speeds in wearable assistive devices. Choosing a more appropriate actuator and gear mechanism could help reduce the power consumption of the RAS.

In terms of ergonomics, the proposed RAS was investigated in end-user tests after integrating the actuation unit in a wearable and portable module. The users perceived the entire hand exoskeleton system, including the RAS, as very lightweight and soft, not resulting in further impediment of their upper-limb range of motion. The overall actuation system is water- and dustproof, sleek, and fulfills the mass requirement of below 1.25 kg to be carried by 6-year-old children with sensorimotors impairment. No adverse events or failures of the RAS occurred during user tests emphasizing safe usage in out-of-the-lab applications. Further, the adaptation of the output of the RAS for thumb actuation of the hand exoskeleton highlights the scalability of the proposed system, paving the way for its use for similar applications in wearable assistive robotics.

The presented RAS allowed significantly higher maximum output force (230 N, increased by factor 1.53) and power-to-mass ratio (10.0 W kg^−1^, increased by factor 4.0) as well as more compact actuators and energy sources (overall power-to-volume ratio of 9.9 kW m^−3^, increased by a factor of 6.19) compared to the RAS previously developed for the hand exoskeleton (Hofmann et al., 2018). The optimized RAS meets the strict requirements for the application with a hand exoskeleton in terms of output force, stroke, and actuation frequency. High power density is a key property for building compact and lightweight RAS but is rarely reported in the literature. Often, output forces and actuator mass are reported while information on output power, volume, and overall mass of the RAS is missing, complicating performance comparison between different approaches and systems. Asbeck et al. (2015) reported output forces of 200 N at a power draw of 59.2 W. For the presented actuator mass of 8.1 kg, a power-to-mass ratio of 7.3 W kg^−1^ could be calculated, however, not taking into account power losses in the actuation, transmission, and potential additional electronics. Schmidt et al. (2017) achieved maximum output forces of 435 N and maximum cable speeds of 0.71 m s^−1^ (cable travel of 200 mm traversed in 280 ms) at an actuator mass of 1.07 kg. Output power and relative power ratios are not reported. In contrast to the application with a hand exoskeleton, the RAS presented in Asbeck et al. (2015) and Schmidt et al. (2017) are intended for lower-limb exosuits, further complicating a direct comparison due to different design requirements for the use cases. Comparing the power density of these mechanical systems to a human muscle (50 W kg^−1^ or higher, Hunter and Lafontaine, 1992), it becomes apparent that there is still a long way to go to meet the performance of the human motor system.

The literature review of existing RAS identified that only a small proportion (31 %) of the reviewed RAS can be considered fully wearable and portable, although targeting applications in fully wearable robotics. In comparison, Chu and Patterson (2018) identified 20 out of the 44 soft robotic devices (45 %) reviewed in their study to be portable. However, no distinction between RAS principles was reported, and portability, as interpreted by Chu and Patterson (2018), does not necessarily mean full wearability, which is a crucial design criterion for out-of-the-lab applications of assistive devices. Compared to other reviews on actuation and transmission systems for wearable assistive devices (Bos et al., 2016; Manna and Dubey, 2018), we additionally investigated and rated ergonomic and maintenance-related aspects of available RAS, thereby possibly providing guidelines supporting other researchers in selecting the most suitable RAS not only for out-of-the-lab applications of wearable assistive devices. Nevertheless, the results of the presented review and evaluation need to be interpreted with caution since the literature research was not conducted systematically, and relevant concepts and guidelines might have been missed. Furthermore, despite our efforts to make the rating process of the different RAS principles as transparent and objective as possible, we acknowledge that this step remains a subjective analysis. In addition, specific advantages and disadvantages of each RAS principle may need to be reconsidered when targeting other applications than fully wearable assistive devices. However, most research developments and industry products rely on cable-based transmissions, suggesting that this is a viable approach for wearable robotics.

Although advances in the development of RAS in terms of durability, ergonomics, and efficiency were achieved in this work, further improvement is required for long-term use in out-of-the-lab applications. To enhance the RAS, the trade-off between flexibility and diameter of the sheaths and cables could be revisited, including the evaluation of additional material pairings. In addition to the limitations of the RAS, the durability evaluation unraveled the next weakest links after the transmission cables in the interconnected chain, which were the Bowden sheath couplers and the rack-and-pinion mechanism at the output that broke after approximately 43,000 and 55,000 cycles, respectively. However, the replacement of broken components during the evaluation process might have led to a bias toward higher rates of failure as wear was carried over the test's restarts. The durability of the broken components should be investigated in more detail. Further research and intensive, longitudinal testing with end-users is required to evaluate functionality and usability of the presented RAS for wearable assistive devices in out-of-the-lab applications, e.g., regarding ergonomics in ADLs or maintenance. In this work, user feedback was collected in a rather unstructured way, mainly from open discussions and focused on the overall system. Collecting feedback from additional end-users and in a more systematic way, e.g., using standardized questionnaires, would allow to draw further conclusions for future development and compare to other researchers' results in the field of wearable assistive robotics.

Overall, by optimizing and evaluating a RAS regarding requirements for out-of-the-lab applications such as durability and ergonomics, we took an essential step toward supporting the transfer of wearable assistive research prototypes from the lab to real-world use.

## Data Availability Statement

The raw data supporting the conclusions of this article will be made available by the authors, without undue reservation.

## Ethics Statement

The studies involving human participants were reviewed and approved by Ethics committee of ETH Zurich (2018-N-90) Ethics committee of the Canton of Zurich (KEK-2019-00409). Written informed consent to participate in this study was provided by the participants' legal guardian/next of kin. Written informed consent was obtained from the individual(s) for the publication of any potentially identifiable images or data included in this article.

## Author Contributions

JD, UH, and TB performed the literature review and the development of the presented remote actuation system. JD performed the test-bench evaluation. All authors revised the article and approved the final version of the manuscript.

## Conflict of Interest

The authors declare that the research was conducted in the absence of any commercial or financial relationships that could be construed as a potential conflict of interest. The handling Editor declared a past collaboration with the authors OL, RG.
