# Supplementary material for: Remote Actuation Systems for Fully Wearable Assistive Devices: Requirements, Selection, and Optimization for Out-of-the-Lab Application of a Hand Exoskeleton
Source: Front Robot AI. 2021 Jan 28;7:596185. doi: 10.3389/frobt.2020.596185 (PMC7876397; doi:10.3389/frobt.2020.596185)
Supplement: Supplementary file 1 [file Data_Sheet_1.PDF]

Supplementary Material

1 SUPPLEMENTARY TABLES AND FIGURES

1.1 Tables

Table S1: Pugh analysis of available fully wearable remote actuation system (RAS) concepts categorized by transmission system for out-of-the-lab applications: arguments for the rating of different RAS concepts were identified from the literature and are listed in the table together with the final scores. The scores were established based on these objective arguments and subjective inputs based on the authors' experience. Abbrevations: Trans. - Transmission, Act. - Actuation, Out. - Output, press. - pressure, dens. - density, controllabil. - controllability, leng. - length.

Trans.	Act.	Out.	Maintenance	Efficiency	Power density	Safety	Ergonomics
Pneumatic	Tank	PAM	−<br>− capacity<br>− refill from air supplies<br>− put on/off<br>− wear & tear	−<br>− air release<br>+ bending<br>− regulators	−<br>− transmission<br>− bulky tanks<br>+ high press. (tanks)<br>− bulky PAM if bidirectional	0<br>+ inherent compliance<br>− buckling	−<br>+ comfort<br>− noise<br>− power dens.<br>− high pressure
	Pump	Bellow	0<br>+ battery<br>− put on/off<br>− wear & tear	0<br>− air release<br>+ bending	−<br>− transmiss.<br>+ lightweight bellow	+<br>+ inherent compliance	0<br>+ comfort
		Cylinder	+<br>+ battery<br>− wear & tear	−<br>− air release<br>+ bending<br>− cylinder eff.	−<br>− transmiss.<br>− stroke leng.<br>− high press. (cylinder)	+<br>+ inherent compliance<br>+ linearity	−<br>− power dens.<br>− high press.
		SPA	0<br>+ battery<br>− put on/off<br>− wear & tear	0<br>− air release<br>+ bending	0<br>− transmiss.<br>+ lightweight SPA	0<br>+ inherent compliance<br>− buckling	0<br>+ comfort

Hydraulic Pump	Bellow	–<br>+ battery<br>– put on/off<br>– wear & tear<br>– leakage	+<br>+ transmiss.	+<br>+ transmiss.<br>+ lightweight bellow	0<br>+ controllab.<br>– hoses burst	+<br>+ comfort<br>+ waterproof<br>– leakage<br>+ power dens.
	Cylinder	–<br>+ battery<br>– wear & tear<br>– leakage	0<br>+ transmiss.<br>– cylinder efficiency	++<br>+ transmiss.<br>+ high press. (cylinder)	–<br>+ linearity<br>– hoses burst<br>– high press.	0<br>+ waterproof<br>– leakage<br>– high press.<br>+ power dens.
	SHA	–<br>+ battery<br>– put on/off<br>– wear & tear<br>– leakage	+<br>+ transmiss.	++<br>+ transmiss.<br>+ lightweight SMA	–<br>– buckling<br>– hoses burst	+<br>+ comfort<br>+ waterproof<br>– leakage<br>+ power dens.
Motor + Cyl.	HAM	–<br>+ battery<br>– put on/off<br>– wear & tear<br>– leakage	+<br>+ transmiss.	0<br>+ transmiss.<br>– stroke length	–<br>– buckling<br>– hoses burst	+<br>+ comfort<br>+ waterproof<br>– leakage
	Cylinder	–<br>+ battery<br>– wear & tear<br>– leakage	0<br>+ transmiss.<br>– cylinder efficiency	+<br>+ transmiss.<br>– stroke leng.<br>+ high press. (cylinder)	–<br>– buckling<br>+ linearity<br>– hoses burst<br>– high press.	–<br>+ waterproof<br>– leakage<br>– high press.
Body-power	Cylinder	0<br>+ no power supply<br>– readjust length	0<br>+ transmiss.<br>– cylinder efficiency	+<br>+ transmiss.<br>– user dependent<br>+ no actuators / power source	+<br>+ direct control	0<br>– wearing comfort<br>– fatigue<br>+ waterproof<br>+ noise

Cable-based	Linear motor	Winch	+<br>+ battery<br>− cables break<br>+ easy to replace	−<br>+ transmiss.<br>− bending<br>− add. friction	+<br>+ compact actuator	+<br>+ compliance<br>− controllabil.<br>+ safe failure	+<br>++ transmission
		Direct	+<br>+ battery<br>− cables break<br>+ easy to replace	0<br>+ transmission<br>− bending	+<br>+ compact actuator	+<br>+ compliance<br>− controllabil.<br>+ safe failure	0<br>+ transmission
	Rotary motor	Winch	+<br>+ battery<br>− cables break<br>+ easy to replace	0<br>+ transmission<br>− bending<br>+ DC motor<br>− add. friction	0<br>+ compact actuator<br>− add. structures	+<br>+ compliance<br>− controllabil.<br>+ safe failure	++<br>++ transmission
		Direct	+<br>+ battery<br>− cables break<br>+ easy to replace	+<br>+ transmission<br>− bending<br>+ DC motor	0<br>+ compact actuator<br>− add. structures	+<br>+ compliance<br>− controllabil.<br>+ safe failure	+<br>+ transmission

1.2 Figures

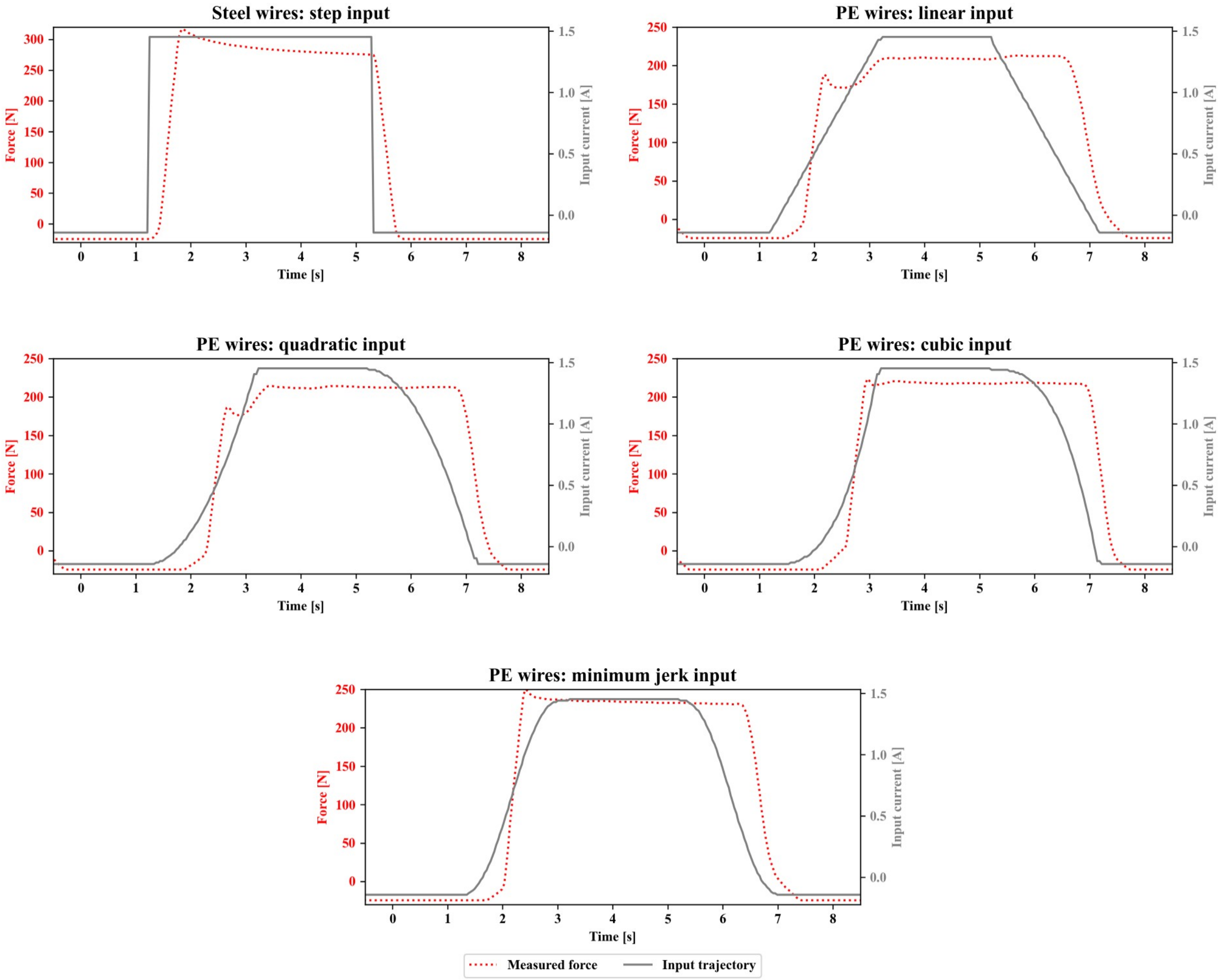

Figure S1: Comparison of the output force profiles of the RAS for different control inputs using PE wires for the transmission: Different control input trajectories were applied to characterize the output force profile. The final current for all input trajectories was 1.46 A. All input trajectories lead to an initial force peak. The output force drops for the linear, quadratic, and cubic input after the initial peak before rising again.

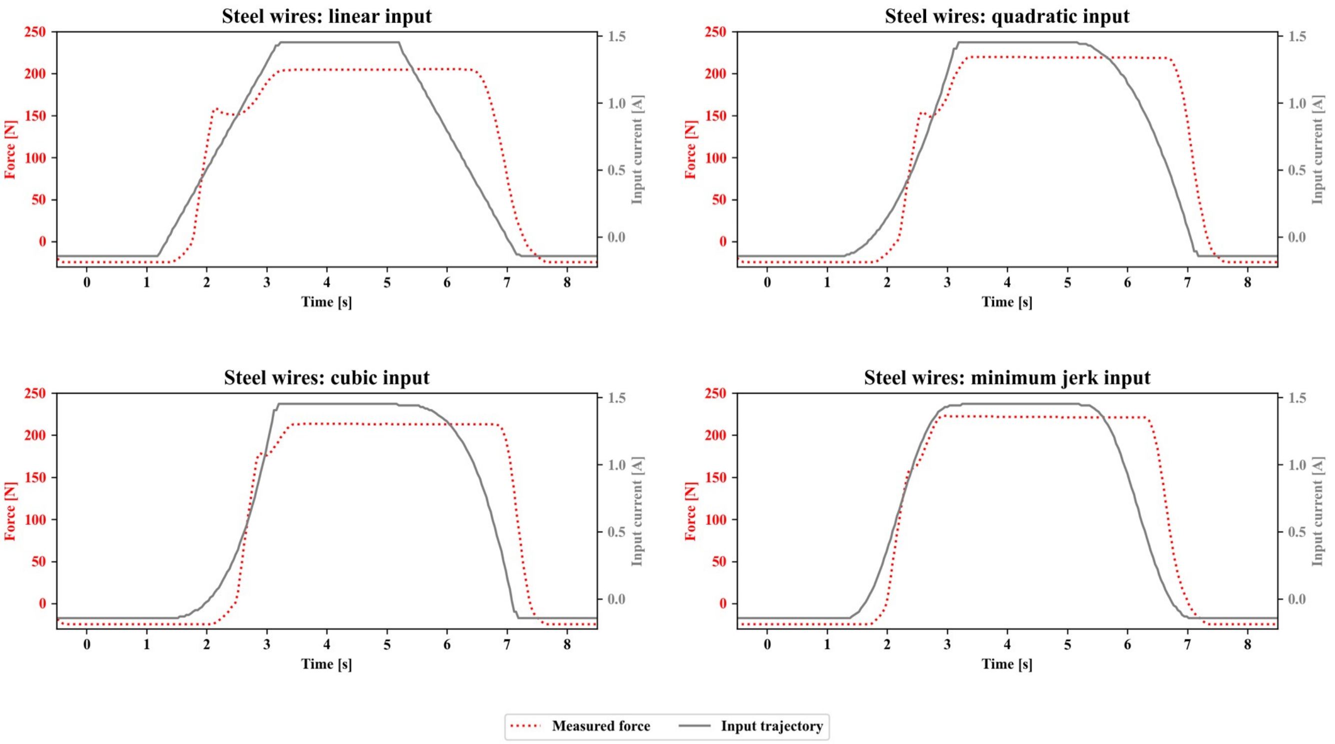

Figure S2: Comparison of the output force profiles of the RAS for different control inputs using steel wires for the transmission: Different control input trajectories were applied to characterize the output force profile. The final current for all input trajectories was 1.46 A. All input trajectories except the minimum jerk lead to an initial force peak. The output force drops for the linear, quadratic, and cubic input after the initial peak before rising again. The output force for a step input is not available since the steel wire tore at lower motor currents than 1.46 A.

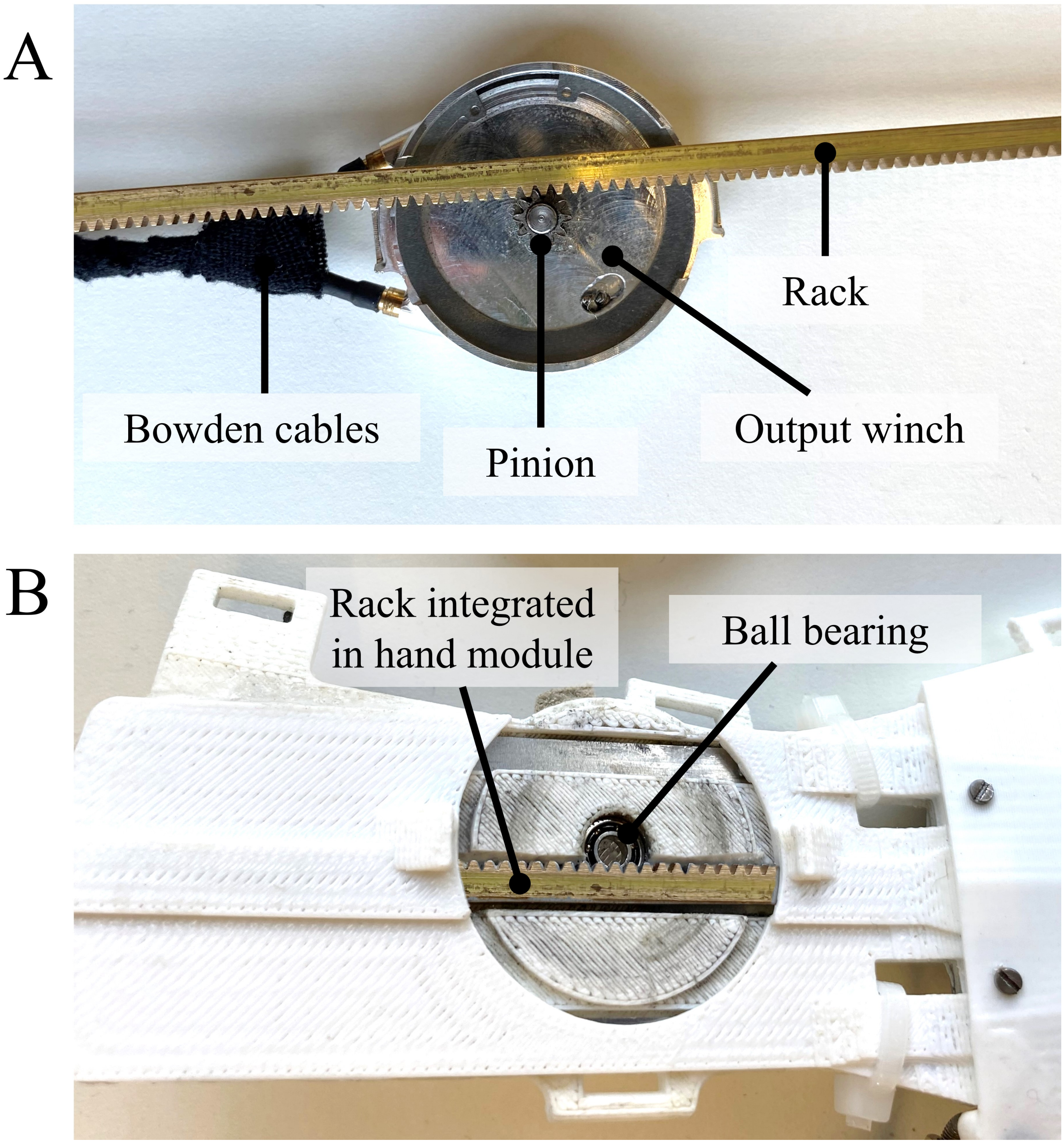

Figure S3: Rack-and-pinion mechanism at the output of the RAS: (A) The torque at the output winch is transmitted to force from a pinion to a rack (B) integrated into the hand exoskeleton.
